# Rac1 Regulates Endometrial Secretory Function to Control Placental Development

**DOI:** 10.1371/journal.pgen.1005458

**Published:** 2015-08-25

**Authors:** Juanmahel Davila, Mary J. Laws, Athilakshmi Kannan, Quanxi Li, Robert N. Taylor, Milan K. Bagchi, Indrani C. Bagchi

**Affiliations:** 1 Department of Comparative Biosciences, University of Illinois at Urbana-Champaign, Urbana, Illinois, United States of America; 2 Department of Molecular and Integrative Physiology, University of Illinois at Urbana-Champaign, Urbana, Illinois, United States of America; 3 Department of Obstetrics and Gynecology, Wake Forest School of Medicine, Winston-Salem, North Carolina, United States of America; Stanford University School of Medicine, UNITED STATES

## Abstract

During placenta development, a succession of complex molecular and cellular interactions between the maternal endometrium and the developing embryo ensures reproductive success. The precise mechanisms regulating this maternal-fetal crosstalk remain unknown. Our study revealed that the expression of Rac1, a member of the Rho family of GTPases, is markedly elevated in mouse decidua on days 7 and 8 of gestation. To investigate its function in the uterus, we created mice bearing a conditional deletion of the *Rac1* gene in uterine stromal cells. Ablation of *Rac1* did not affect the formation of the decidua but led to fetal loss in mid gestation accompanied by extensive hemorrhage. To gain insights into the molecular pathways affected by the loss of *Rac1*, we performed gene expression profiling which revealed that Rac1 signaling regulates the expression of Rab27b, another GTPase that plays a key role in targeting vesicular trafficking. Consequently, the *Rac1*-null decidual cells failed to secrete vascular endothelial growth factor A, which is a critical regulator of decidual angiogenesis, and insulin-like growth factor binding protein 4, which regulates the bioavailability of insulin-like growth factors that promote proliferation and differentiation of trophoblast cell lineages in the ectoplacental cone. The lack of secretion of these key factors by *Rac1*-null decidua gave rise to impaired angiogenesis and dysregulated proliferation of trophoblast cells, which in turn results in overexpansion of the trophoblast giant cell lineage and disorganized placenta development. Further experiments revealed that *RAC1*, the human ortholog of *Rac1*, regulates the secretory activity of human endometrial stromal cells during decidualization, supporting the concept that this signaling G protein plays a central and conserved role in controlling endometrial secretory function. This study provides unique insights into the molecular mechanisms regulating endometrial secretions that mediate stromal-endothelial and stromal-trophoblast crosstalk critical for placenta development and establishment of pregnancy.

## Author Summary

During early pregnancy, a succession of molecular interactions between the uterus and the developing embryo ensures reproductive success. Although it is conceivable that signaling cues originating in the uterus impact on the developing embryo at the time of placenta establishment, the precise mechanisms regulating the maternal-fetal crosstalk remain unknown. Impaired uterine functions during early pregnancy are likely to contribute to abnormal embryo development and various diseases of pregnancy, such as recurrent miscarriage, preeclampsia, and intrauterine growth restriction. This study provides insights into the molecular mechanisms by which Rac1, a signaling molecule expressed in the decidua, controls uterine secretions that mediate maternal-fetal communication critical for placental development and establishment of pregnancy.

## Introduction

Shortly after fertilization, the uterus transitions to a receptive state that allows embryo attachment and invasion, and this process must be synchronized with embryonic development in order to ensure maximal reproductive success [1–5]. To enable this synchronization, an intricate maternal-fetal dialogue has evolved that allows the developing embryo and the uterus to be in constant communication with each other. In humans and rodents, as pregnancy progresses, the uterus undergoes a dramatic transformation to form the decidua, a stroma-derived secretory tissue that encases the growing fetus for the duration of pregnancy [3–6]. Decidual cells are responsible for producing and secreting paracrine factors that promote the formation of an extensive vascular network that supports embryo development [7–9]. Proper differentiation and migration of the trophoblast cells, critical for the formation of a functional placenta, are also influenced by as yet unknown factors secreted by the differentiating stromal cells. If any of these processes fail to proceed normally, a number of diseases of pregnancy can result, such as recurrent miscarriage, preeclampsia, and intrauterine growth restriction [10–12]. The current challenge is to understand the complex processes by which various signaling molecules emanating from the maternal decidua communicate with trophoblasts to ensure successful establishment and maintenance of pregnancy.

In this study, using genetic and cell biological approaches, we demonstrate that Ras-related C3 botulinum toxin substrate 1 (Rac1), a maternal factor expressed in decidual cells, regulates the secretory pathways that mediate stromal-endothelial and stromal-trophoblast crosstalk within a narrow temporal window during placenta development. Rac1 belongs to the Rho family of GTPases and is a key signaling molecule that regulates cell proliferation, differentiation, cell-cell adhesion, and cell motility [13–16]. It controls these processes by acting as a G protein, a molecular switch that becomes active when bound to GTP or inactive when bound to GDP [13–16]. Our studies revealed that Rac1 expression is induced in decidualizing stromal cells following implantation. Conditional ablation of endometrial *Rac1* led to a severe defect in fertility. Further analysis revealed that uteri lacking *Rac1* are able to undergo decidualization as indicated by weight gain assay and the expression of biochemical markers of this process. However, in the absence of Rac1, the expression of Rab27b, another G protein that plays a key role in vesicular exocytosis [17, 18], is markedly impaired in the decidual cells. Consistent with this finding, our studies revealed that the *Rac1*-null decidual cells exhibit a defect in the secretion of vascular endothelial growth factor A (VEGFA) and insulin-like growth factor binding protein 4 (IGFBP4). Deficiency of VEGFA in *Rac1*-null uteri contributed to impaired decidual angiogenesis, while the lack of action of IGFBP4 was associated with dysregulated expansion and differentiation of trophoblast cells, resulting in disorganized placenta formation and pregnancy failure. Further studies revealed that *RAC1*, the human ortholog of *Rac1*, regulates the secretion of VEGFA by primary human endometrial stromal cells during decidualization, highlighting its conserved role in regulating endometrial secretory function. Collectively, our study provides important insights into the molecular mechanisms that control endometrial secretions mediating stromal-endothelial and stromal-trophoblast crosstalk critical for placental development and establishment of pregnancy.

## Results

### Induction of Rac1 expression and activation in the decidua

**Fig 1:**
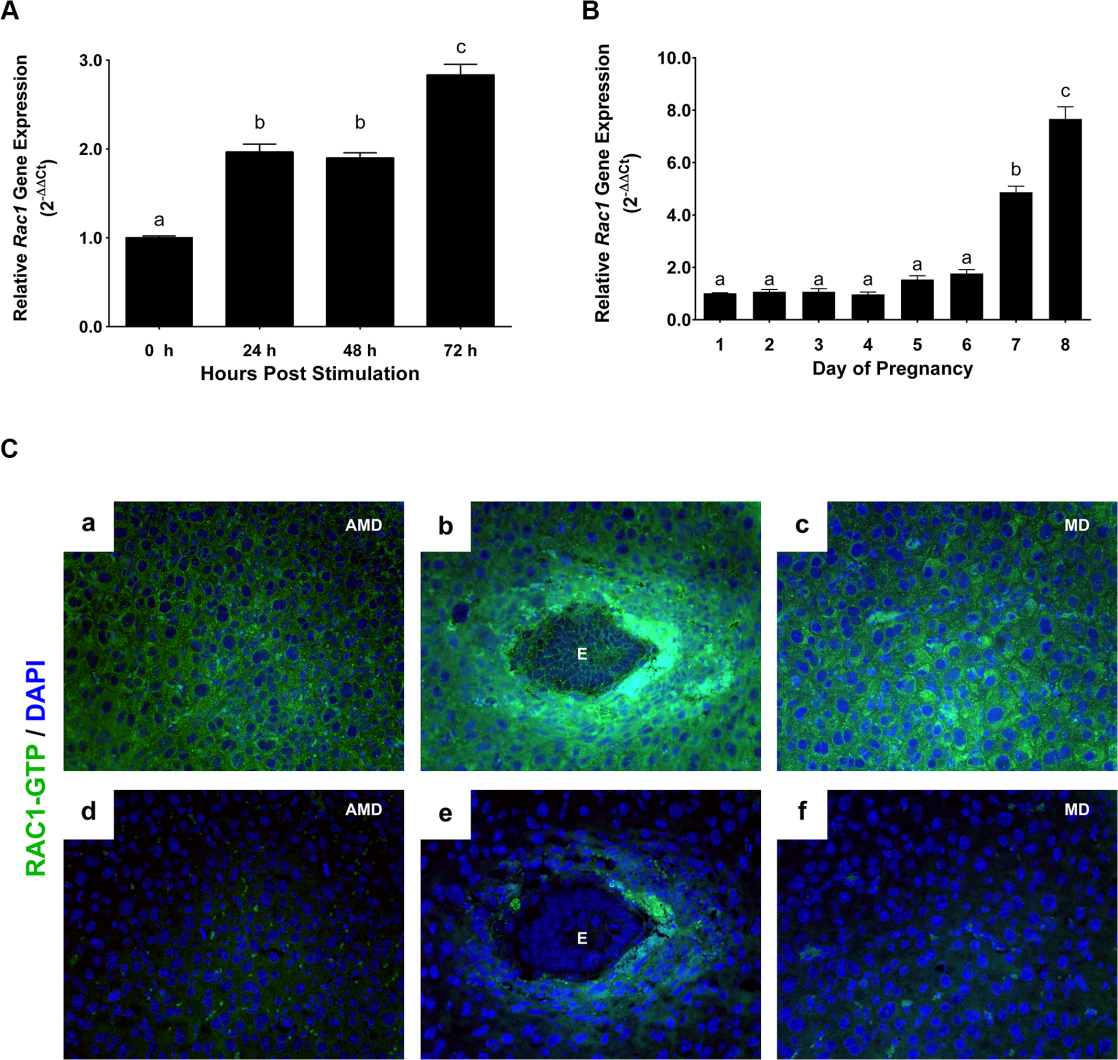
Rac1 is induced in the uterus during early pregnancy. **(A)** Induction of *Rac1* mRNA in the uterus during experimentally-induced decidualization. Uterine RNA was purified from mice at different times after decidual stimulation and analyzed by qPCR. Relative levels of *Rac1* mRNA expression in uteri after decidual stimulation are compared to those in unstimulated control uteri. Data represent mean ± SEM from three separate samples and were analyzed by one-way ANOVA with Bonferroni post-test. Letters indicate statistically significant differences (*P* < 0.0001). **(B)** Expression of *Rac1* during early pregnancy overlaps with the decidual phase of gestation. qPCR was performed to monitor the expression of *Rac1* mRNA in uteri on days 1 to 8 of gestation. The relative levels of gene expression on different days of pregnancy were determined by setting the expression level of *Rac1* mRNA on day 1 of pregnancy at 1.0. *Rplp0*, encoding a ribosomal protein, was used to normalize the level of RNA. Data represent mean ± SEM from three separate samples and were analyzed by one-way ANOVA with Bonferroni post-test. Letters indicate statistically significant differences (*P* < 0.0001). **(C)** Localization of active RAC1 protein in uterine stromal cells during early pregnancy. Uterine sections on day 7 of pregnancy were subjected to immunofluorescence (IF) histochemistry using anti-RAC1-GTP antibody. Panels a, b, and c show immunostaining of RAC1-GTP; panels d, e, and f show staining with non-immune IgG. AMD, MD and E indicate antimesometrial decidua, mesometrial decidua and embryo, respectively.

To gain insights into the molecular pathways underlying decidualization, we performed gene expression profiling to analyze alterations in uterine gene expression patterns in response to decidual stimulation. In rodents, decidualization can be induced experimentally in the absence of an implanting embryo [19, 20]. In this protocol, non-pregnant ovariectomized mice are primed with steroid hormones and decidualization is initiated in one uterine horn by injecting oil, which mimics implanting blastocysts, while the other horn is left untreated and serves as a control. As shown previously, a robust decidual response is seen in the oil-stimulated horn by 72 h, whereas no decidualization occurs in the unstimulated horn [20]. Gene expression profiling, using RNA isolated from stimulated and unstimulated uterine horns identified many genes whose expression was significantly altered in the uterus in response to decidual stimulation (GEO accession GSE70572). Ingenuity Pathway Analysis revealed that the genes associated with cell-cell signaling, metabolism, extracellular matrix and integrin signaling, angiogenesis, and signaling by the TGFβ family and WNTs constitute the biological categories mostly affected in response to decidual stimulation. Among these factors, we focused on *Rac1* because a previous *in vitro* study implicated that RAC1 plays a critical role during implantation in the human [21]. To confirm the results of the microarray analysis, we performed qPCR. As shown in Fig 1A, maximal *Rac1* transcript levels were observed 72 h after decidual stimulation. Consistent with this finding, we observed a significant up regulation of *Rac1* transcripts during early pregnancy on days 7 and 8 of normal mouse gestation (Fig 1B).

Rac1, a G protein, controls downstream signaling pathways by acting as a molecular switch that becomes active when bound to GTP [13, 15]. To determine whether the active form of Rac1 protein is present in the decidual uterus, we analyzed uterine sections on day 7 of pregnancy by performing immunofluorescence histochemistry using an antibody that specifically recognizes Rac1-GTP. We observed intense expression of active Rac1 protein in decidual cells surrounding the implanted embryo and also in the mesometrial and antimesometrial decidua (Fig 1C).

### Conditional deletion of *Rac1* in the endometrium leads to severe infertility

**Fig 2:**
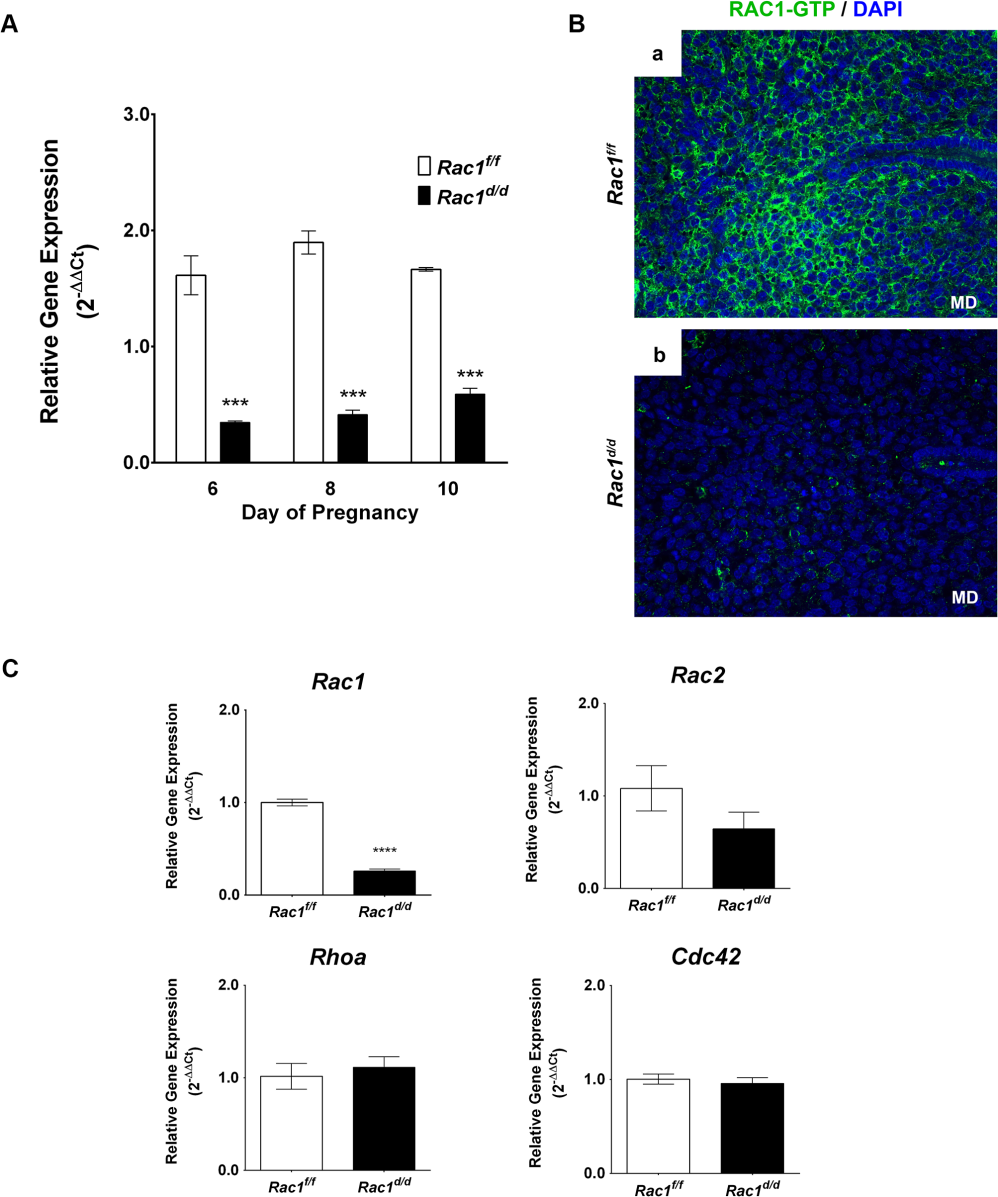
Loss of Rac1 expression in the uterus of *Rac1* ^*d/d*^ mice. **(A)** Efficient ablation of *Rac1* in the uterus during decidual phase of pregnancy. Uterine RNA was purified from *Rac1* ^*f/f*^ and *Rac1* ^*d/d*^ mice on days 6, 8, or 10 of pregnancy and analyzed by qPCR. Relative levels of *Rac1* mRNA expression in uteri of *Rac1* ^*d/d*^ mice are compared to those in *Rac1* ^*f/f*^ control mice. Data represent mean ± SEM from four separate samples and were analyzed by two-way ANOVA with Bonferroni post-test. Asterisks indicate statistically significant differences (***P < 0.001) **(B)** A marked decline in the level of active RAC1 protein in the stromal cells of *Rac1* ^*d/d*^ uteri. Uterine sections obtained from day 8 pregnant *Rac1* ^*f/f*^ (left panel) and *Rac1* ^*d/d*^ (right panel) mice were subjected to IF using anti-RAC1-GTP antibody. Note the lack of RAC1 immunostaining in uteri of the mutant mice. **(C)** Expression of various members of the Rho family of GTPases was unaffected in *Rac1* ^*d/d*^ uteri. Real-time PCR was performed to monitor the expression of *Rac1*, *Rac2*, *Rhoa*, and *Cdc42* in the uteri of day 8 pregnant *Rac1* ^*f/f*^ and *Rac1* ^*d/d*^ mice. Data represent mean ± SEM from four separate samples and were analyzed by *t*-test. Asterisks indicate statistically significant differences (****P < 0.0001).

To investigate the function of *Rac1* in the uterus, we conditionally deleted *Rac1* gene in the uteri of adult mice. The conditional deletion approach was used because the global knockout of *Rac1* causes embryonic lethality [22]. We crossed mice harboring the “floxed” *Rac1* [23] (*Rac1* ^*f/f*^) with *Pgr* ^*Cre/+*^ mice to create *Rac1* ^*d/d*^ mice. This approach was previously used by several laboratories to ablate “floxed” genes selectively in cells expressing PGR (progesterone receptor), including uterine cells [20, 24–27]. We assessed the extent of deletion of *Rac1* in the uteri of *Rac1* ^*d/d*^ mice by qPCR and immunofluorescence (Fig 2). Our results showed greatly reduced expression of *Rac1* transcripts in the uteri on days 6 to 10 of pregnancy, indicating efficient ablation of *Rac1* gene in the uteri of *Rac1* ^*d/d*^ mice (Fig 2A). Consistent with the RNA profile, we observed a marked decline in the levels of active Rac1 protein in *Rac1* ^*d/d*^ uteri on day 8 of gestation (Fig 2B). We further noted that the expression of other members of the Rho family of GTPases, including *Rac2*, *Rhoa*, and *Cdc42*, was unaffected in *Rac1* ^*d/d*^ uteri (Fig 2C).

A six-month breeding study was performed by crossing *Rac1* ^*d/d*^ or *Rac1* ^*f/f*^ females with wild-type males of proven fertility (Table 1). This breeding scheme was employed so that the implanting embryos in *Rac1*-deficient uteri are either intact or heterozygous at the *Rac1* gene locus. At the completion of the study, we noted more than 90% reduction in the total number of pups born to *Rac1* ^*d/d*^ dams compared with the control *Rac1* ^*f/f*^ females (Table 1). The females heterozygous for the *Rac1* gene delivered the same number of pups as that of *Rac1* ^*f/f*^ females. These results indicated that the severe fertility defect is attributable to the lack of Rac1 expression in PGR expressing uterine cells of *Rac1* ^*d/d*^ females.

**Table 1:** Ablation of uterine *Rac1* leads to severe female infertility.

Genotype	Number of Females^†^	Number of Pups	Number of Litters	Average Pups/Litter^‡^
***Rac1^f/f^***	**7**	**307**	**31**	**10**	
***Rac1^d/d^***	**7**	**19**	**11**	**2**	

^†^ Some *Rac1* ^*d/d*^ females did not deliver any pups during or after the breeding study

^‡^ *P* < 0.0001

We next investigated whether the infertility of *Rac1* ^*d/d*^ females was due to an ovarian defect. Ovaries from *Rac1* ^*f/f*^ and *Rac1* ^*d/d*^ females on days 4, 8, and 12 of pregnancy were collected, and evaluated histologically for the presence of corpora lutea (CL). As shown in S1A Fig, ovaries collected from *Rac1* ^*f/f*^ and *Rac1* ^*d/d*^ females displayed comparable histology with follicles at all stages of development and CL with normal appearance. To examine ovulation and fertilization in these mice, blastocysts were recovered from uteri of *Rac1* ^*f/f*^ and *Rac1* ^*d/d*^ mice on day 4 of pregnancy prior to implantation. No significant difference was found either in the morphology or number of the embryos recovered from *Rac1* ^*f/f*^ and *Rac1* ^*d/d*^ uteri (S1B Fig). In further support of normal ovarian activity, we noted comparable levels of serum progesterone in *Rac1* ^*f/f*^ and *Rac1* ^*d/d*^ females on days 4, 8, 10, and 12 of pregnancy (S1C Fig). Collectively, these results indicated that the infertility of *Rac1* ^*d/d*^ females is not due to impairment in the hypothalamic-pituitary-ovarian axis or lack of fertilization but is likely due to defective uterine function.

### Embryo attachment and decidualization are unaffected in *Rac1* ^*d/d*^ mice

**Fig 3:**
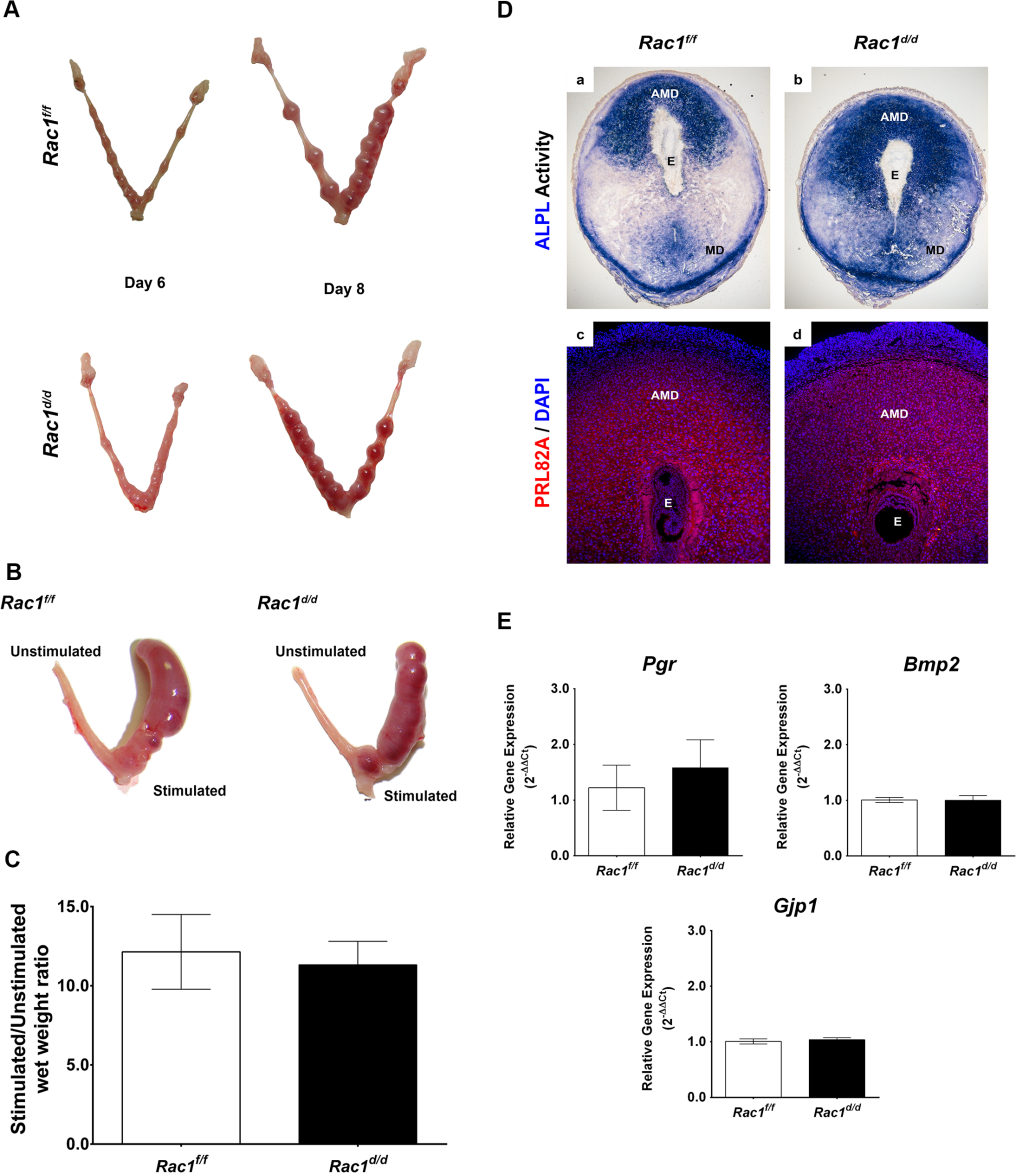
Early implantation is unaffected in *Rac1* conditional knockout mouse. **(A)** Gross morphology of *Rac*1^*f/f*^ and *Rac*1^*d/d*^ uteri at days 6 and 8 of gestation. **(B)** *Rac*1^*f/f*^ and *Rac*1^*d/d*^ mice were subjected to artificial decidual stimulation for 96 hours as described in the Materials and methods. For each mouse, one uterine horn was stimulated, while the other horn was left undisturbed. Gross morphology of *Rac*1^*f/f*^ and *Rac*1^*d/d*^ uteri following the application of the decidual stimulus is shown. **(C)** Comparative wet weight gains in uteri of *Rac*1^*f/f*^ and *Rac*1^*d/d*^ mice. Following artificial decidualization, stimulated and unstimulated horns were assessed for wet weight gain. The histogram shows the ratios of average weights of stimulated over unstimulated horns from *Rac*1^*f/f*^ and *Rac*1^*d/d*^ mice. Data represent mean ± SEM from four separate samples and were analyzed by *t*-test, *P > 0*.05). **(D)** Uterine sections from *Rac*1^*f/f*^ and *Rac*1^*d/d*^ mice on day 8 of pregnancy were subjected to alkaline phosphatase activity (ALPL, upper) and IF staining using an antibody specific for the prolactin-related protein (PRL82A, lower). AMD, MD, and E denote antimesometrial decidua, mesometrial decidua, and embryo respectively. **(E)** Comparable expressions of various markers of decidualization in *Rac*1^*f/f*^ and *Rac*1^*d/d*^ uteri. Total RNA was isolated from uteri on day 8 of pregnancy and qPCR analysis was performed using primers specific for *Pgr*, *Bmp2*, and *Gja1*. Data represent mean ± SEM from four separate samples and were analyzed by *t*-test, *P > 0*.05).

Gross examination of uterine morphology revealed apparently normal embryonic implantation sites in *Rac1* ^*f/f*^ and *Rac1* ^*d/d*^ uteri on days 6 and 8 of pregnancy (Fig 3A). There was no apparent defect in uterine receptivity, embryo attachment and formation of decidual mass in pregnant *Rac1* ^*d/d*^ uteri. To further analyze the decidual response in *Rac1* ^*d/d*^ females, we performed experimentally induced decidualization. As shown in Fig 3B, both *Rac1* ^*f/f*^ and *Rac1* ^*d/d*^ uteri exhibited robust decidual responses upon stimulation. When the decidual responses were assessed by measurement of uterine wet weight gain, there was no significant difference between *Rac1* ^*f/f*^ and *Rac1* ^*d/d*^ uteri (Fig 3C). Consistent with these observations, the expression of prolactin-related protein (PRL8A2/dPRP) and alkaline phosphatase (ALPL), known biomarkers of decidualization [28–31] was comparable in the uterine sections of *Rac1* ^*f/f*^ and *Rac1* ^*d/d*^ mice on day 8 of pregnancy (Fig 3D). We additionally examined the expression of a panel of factors, Pgr, Bmp2 and Gja1 (Cx43), which are known regulators of decidualization in mice [20, 25, 31, 32]. Our studies showed that the expression of *Pgr*, *Bmp2*, and *Gja1* mRNAs remained unaffected by the loss of uterine Rac1, indicating that at least certain aspects of the decidualization process progresses normally in *Rac1* ^*d/d*^ uteri (Fig 3E).

### Pregnancy failure in *Rac*1^*d/d*^ mice in mid gestation is associated with failed angiogenesis

**Fig 4:**
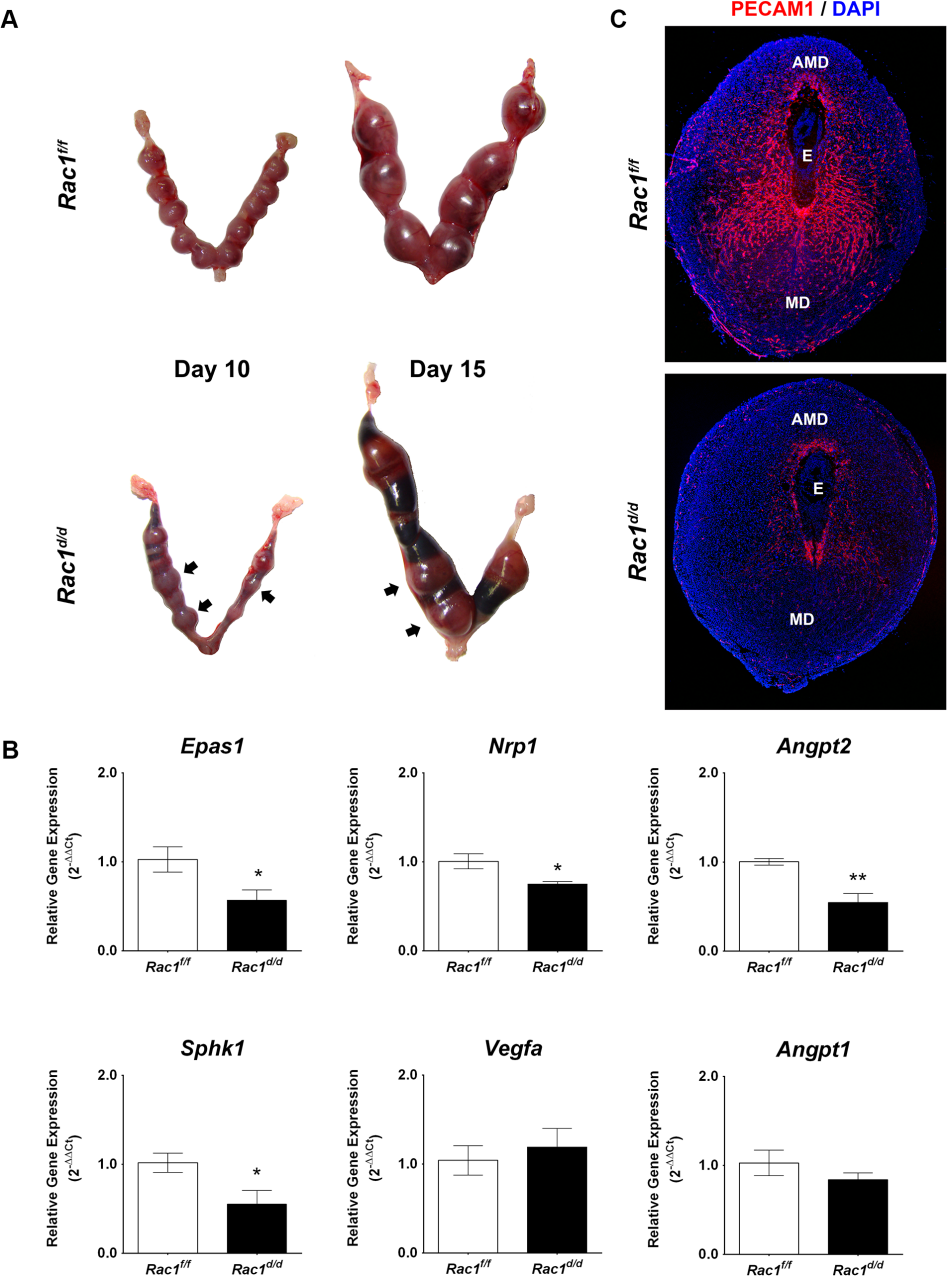
Pregnancy failure in *Rac*1^*d/d*^ mice in mid gestation is associated with lack of angiogenesis. **(A)** Gross morphology of *Rac*1^*f/f*^ and *Rac*1^*d/d*^ uteri at days 10 and 15 of gestation. Hemorrhagic sites are indicated by arrowheads. **(B)** Angiogenesis is impaired in *Rac1* conditional-knockout mouse. qPCR was performed to analyze the expression of angiogenic factors, *Epas1/Hif2α*, *Nrp1*, *Angpt2*, *Sphk1*, *Vegfa*, and *Angpt1* in uteri of *Rac*1^*f/f*^ and *Rac*1^*d/d*^ mice on day 8 of pregnancy. Data represent mean ± SEM from four separate samples and were analyzed by *t*-test. Asterisks indicate statistically significant differences (*P < 0.05, **P < 0.01). **(C)** Uterine sections of *Rac*1^*f/f*^ and *Rac*1^*d/d*^ mice on day 8 were subjected to IF staining with PECAM1 antibody. AMD, MD, and E denote antimesometrial decidua, mesometrial decidua, and embryo respectively

Although no apparent functional abnormality was detected in pregnant *Rac1* ^*d/d*^ uteri up to day 8 of gestation, we observed distinct signs of hemorrhage and embryo resorption in these uteri starting on day 10. By day 15 of gestation, most of the embryos were resorbed in *Rac1* ^*d/d*^ uteri (Fig 4A). To investigate the biological pathways affected by *Rac1* deletion in the uterus, we performed gene expression profiling. Microarray analysis of decidual tissues isolated from *Rac1* ^*f/f*^ and *Rac1* ^*d/d*^ uteri on day 8 of pregnancy revealed downregulation of mRNAs corresponding to many genes among which those controlling vascular development, metabolic processes, cell differentiation, cell adhesion, and vesicular trafficking were prominent (GEO accession GSE70446). Because of the vascular defect and hemorrhage in *Rac1* ^*d/d*^ uteri, we focused on the angiogenesis-related pathways and found several factors, including *Angpt2*, *Nrp1*, *Sphk1*, and *Epas1/Hif2α*, which were downregulated in *Rac1* ^*d/d*^ uteri compared to *Rac1* ^*f/f*^ uteri as early as day 8 of pregnancy (S1 Table). Consistent with the microarray data, qPCR experiments validated that the expression of these factors were indeed markedly reduced, while the expression of several other angiogenic factors, such as *Vegfa*, *Hif1α*, *Angpt1*, *Egln1*, and their receptors, remained unaltered in *Rac1* ^*d/d*^ uteri (Figs 4B and S2A).

We next examined the development of vascular networks in pregnant uteri of *Rac1* ^*d/d*^ mice by employing immunofluorescence using an antibody against platelet/endothelial cell adhesion molecule 1 (PECAM1), a marker of endothelial cells. Uterine sections of the control *Rac1* ^*f/f*^ mice on day 8 of pregnancy exhibited a well-developed vascular network that spreads throughout the decidual bed surrounding the implanted embryo (Fig 4C). In contrast, the PECAM1 immunostaining was markedly reduced in uterine sections of pregnant *Rac1* ^*d/d*^ mice, indicating impaired development of uterine vasculature in the absence of Rac1 signaling (Fig 4C). This reduced angiogenesis was associated with considerable hemorrhagic activity in the implantation chambers of *Rac1* ^*d/d*^ mice. Staining of uterine sections of these mice with eosin-Y, which labels red blood cells (RBCs), confirmed that the RBCs have extravasated from the lateral sinusoids (S2B Fig).

### Abnormal trophoblast proliferation and differentiation in *Rac*1^*d/d*^ uteri

**Fig 5:**
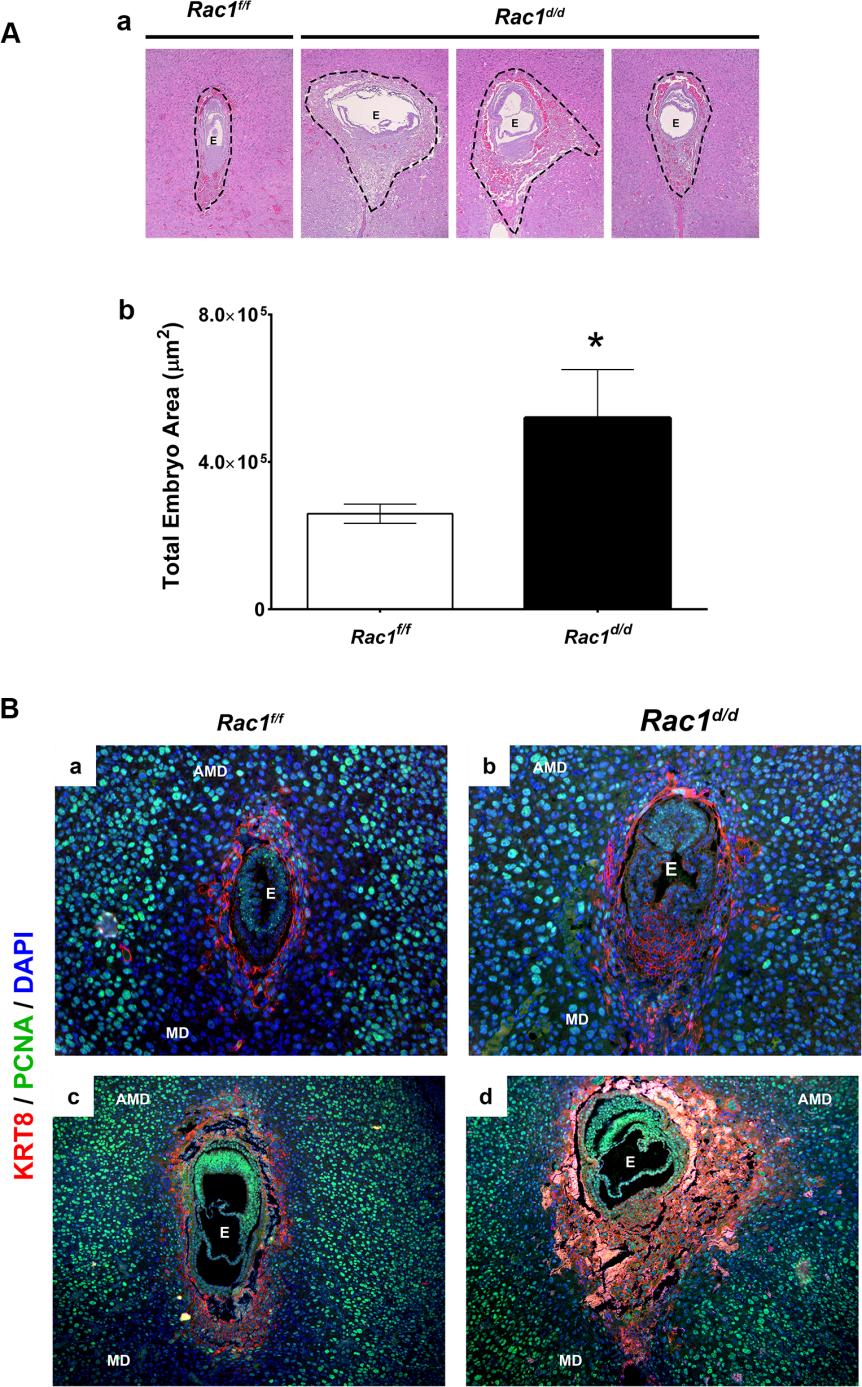
Enhanced trophoblast proliferation in *Rac1* conditional-knockout mouse. **(A)** Expanded trophoblast cells in *Rac*1^*d/d*^ uteri on day 8 of gestation. **a:** Hematoxylin and Eosin (H & E) staining of uterine sections from *Rac1* ^*f/f*^ and *Rac1* ^*d/d*^ mice on day 8 of pregnancy. Three representative images of *Rac*1^*d/d*^ uterine sections are shown. **b:** Quantitation of embryonic areas in H & E stained uterine sections of *Rac1* ^*f/f*^ and *Rac1* ^*d/d*^ mice on day 8 of pregnancy. Data represent mean ± SEM from six separate samples and were analyzed by non-parametric *t*-test. Asterisks indicate statistically significant differences (*P < 0.05). **(B)** Increased proliferation of trophoblast cells in the ectoplacental cone (EPC) of *Rac*1^*d/d*^ uteri. Uterine sections from *Rac1* ^*f/f*^ and *Rac1* ^*d/d*^ mice on days 7 (panels a and b) and 8 of pregnancy (panels c and d) were subjected to IF using PCNA and cytokeratin 8 (KRT8) antibodies. AMD, MD, and E denote antimesometrial decidua, mesometrial decidua, and embryo respectively.

*Rac1*-null uteri exhibited embryo resorption on day 10 of pregnancy, but a closer histological examination of uterine sections revealed that embryos implanted in these uteri begin to show abnormalities as early as days 7–8 of gestation. Specifically, when we measured embryonic areas, we observed a significant expansion of the trophoblast cell layer in the embryos implanted in *Rac1* ^*d/d*^ uteri compared to those in *Rac1* ^*f/f*^ controls (Fig 5A). Further analyses of uterine sections on days 7 and 8 of pregnancy, using antibodies against PCNA, a cell proliferation marker, confirmed enhanced proliferation of the trophoblast cells, marked by cytokeratin 8 staining, within the ectoplacental cone (EPC) of *Rac1* ^*d/d*^ uteri (Fig 5B). We noted with interest that the timeframe of development of this embryonic phenotype in *Rac1*-null uteri closely overlaps with that of induction of *Rac1* expression in the endometrial stromal cells during decidualization.

**Fig 6:**
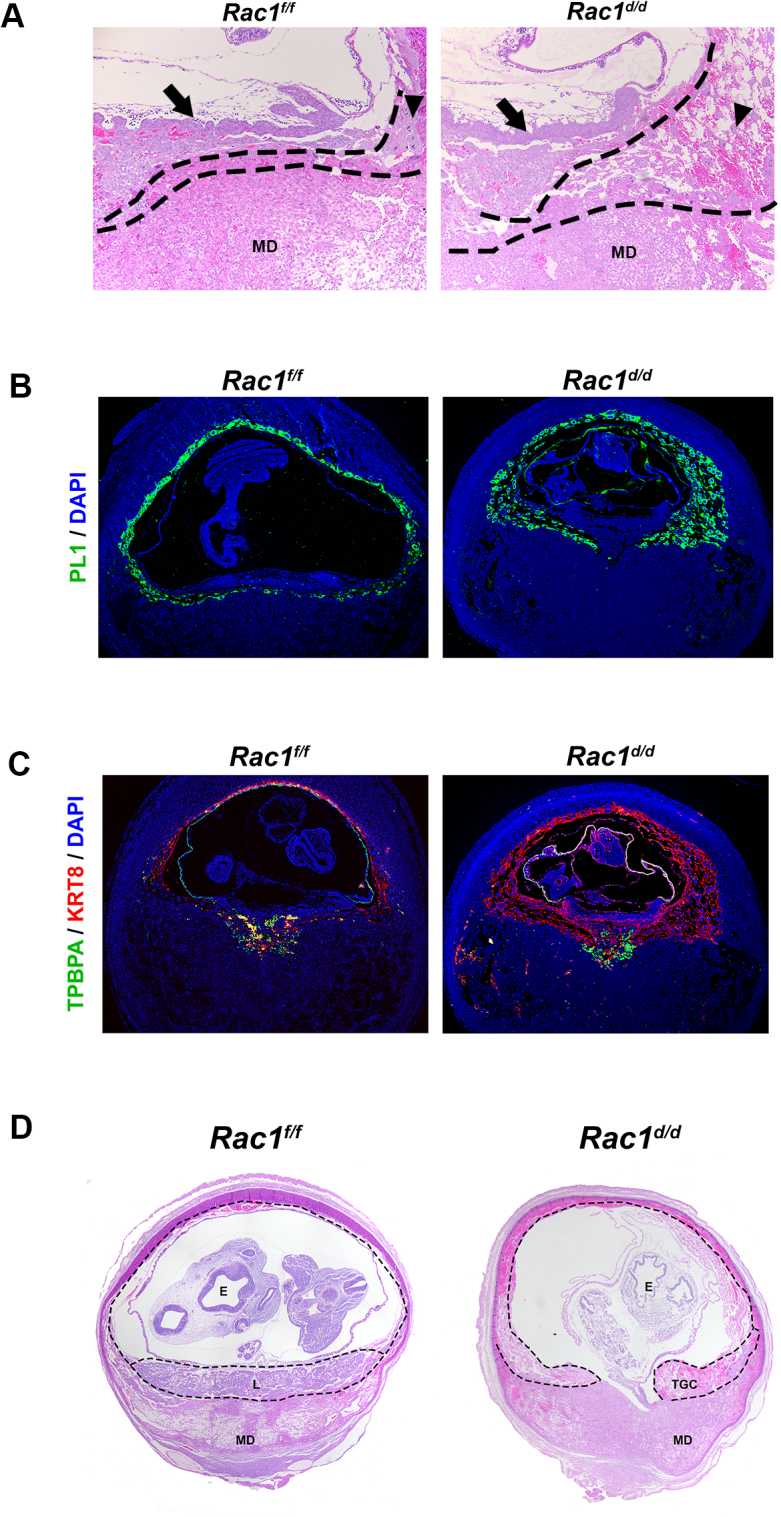
Abnormal trophoblast proliferation, differentiation and disorganized placentation in *Rac1* conditional-knockout mouse. (A & B) Increased population of trophoblast giant cells (TGCs) in the placenta of *Rac*1^*d/d*^ uteri. **(A)** H and E staining of uterine sections from *Rac1* ^*f/f*^ and *Rac1* ^*d/d*^ mice on day 10 of pregnancy. TGC and MD indicate trophoblast giant cells and mesometrial decidua, respectively. TGCs are demarcated by dashed lines. Arrows indicate chorionic plate and arrow-heads indicate TGCs. **(B)** Uterine sections from *Rac1* ^*f/f*^ and *Rac1* ^*d/d*^ mice on day 10 of pregnancy were subjected to IF using PL1. (**C**) Uterine sections from *Rac1* ^*f/f*^ and *Rac1* ^*d/d*^ mice on day 10 of pregnancy were subjected to IF using TPBPA and KRT8 antibodies. (**D)** H and E staining of uterine sections from *Rac1* ^*f/f*^ and *Rac1* ^*d/d*^ mice on day 12 of pregnancy. TGC, MD and E indicate trophoblast giant cells, mesometrial decidua, and embryo, respectively.

To assess the impact of dysregulated proliferation of trophoblast cells in the EPC on placenta formation, we performed histological analyses of uterine sections of *Rac1* ^*d/d*^ and *Rac1* ^*d/d*^ mice on day 10 of gestation. As expected, the placentae of *Rac1* ^*f/f*^ mice displayed normal characteristics of one to two layers of trophoblast giant cells (TGC) at the maternal-fetal interface on day 10. In contrast, we observed up to four or five layers of TGCs in *Rac1* ^*d/d*^ animals (Fig 6A). The abnormal expansion of the TGCs in the placentae of *Rac1* ^*d/d*^ mice was further confirmed when we subjected uterine sections to immunofluorescence analyses using antibodies against PL1, a TGC-specific marker [33–35]. Consistent with the results shown in Fig 6A, multiple layers of TGCs were evident at the maternal-fetal interface in *Rac1* ^*d/d*^ mice compared to one or two layers in *Rac1* ^*f/f*^ mice (Fig 6B). The spatial distribution of the spongiotrophoblast cells, a subtype of TGCs, as indicated by the expression of their biomarker TPBPA [33–35], was identical in the placentae of *Rac1* ^*f/f*^ and *Rac1* ^*d/d*^ mice (Fig 6C). As pregnancy progressed to day 12, the *Rac1* ^*d/d*^ placentae appeared to be highly disorganized, lacking properly formed layers, including the labyrinth (Fig 6D). Taken together, our results indicated that decidual expression of Rac1 critically controls the proliferation and differentiation of the TGCs at the maternal-fetal interface and ensures proper placenta development and structure.

### Rac1 controls Rab27b expression in decidual cells

**Fig 7:**
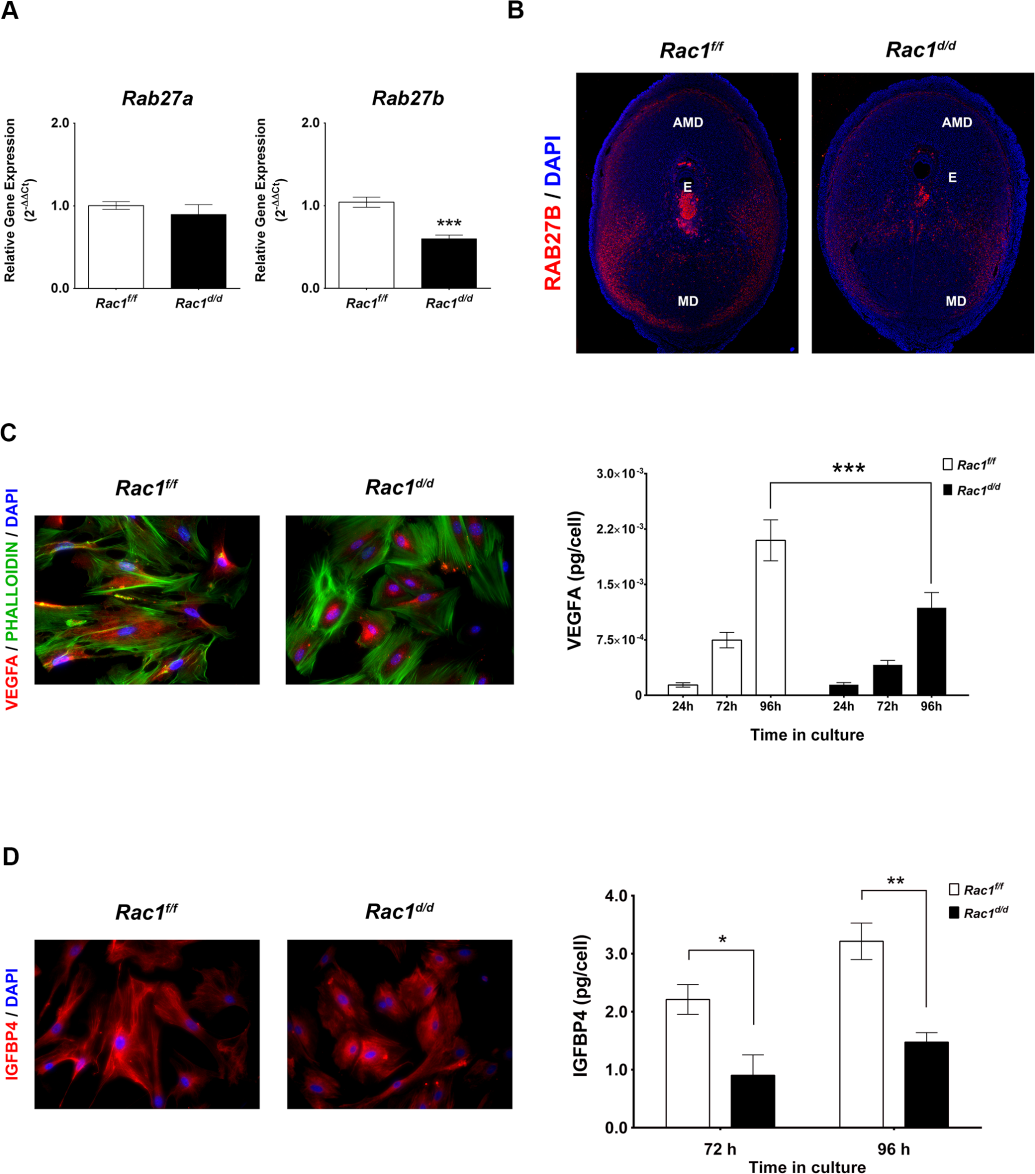
Rac1 regulates vesicular exocytosis in decidual cells by controlling Rab27b. **(A & B)** Expression of Rab27b mRNA and protein is downregulated in Rac1-null stromal cells. **(A)** qPCR was performed to monitor the expression of *Rab27a* and *Rab27b* in the uteri of *Rac*1^*f/f*^ and *Rac*1^*d/d*^ mice on day 8 of pregnancy. Data represent mean ± SEM from four separate samples and were analyzed by *t*-test. Asterisks indicate statistically significant differences (***P < 0.001). **(B)** IF of RAB27B in *Rac1* ^*f/f*^ and *Rac1* ^*d/d*^ uteri on day 8 of pregnancy. AMD, MD, and E denote antimesometrial decidua, mesometrial decidua, and embryo respectively. **(C & D)** Secretions by decidual cells are reduced in the conditioned media of Rac1-null stromal cells. Stromal cells isolated from *Rac1* ^*f/f*^ and *Rac1* ^*d/d*^ uteri on day 4 of pregnancy were cultured for 96 hours, fixed and subjected to IF using VEGFA (**C, Left**) and IGFBP4 (**D, Left**) antibodies. Conditioned media from cultured stromal cells isolated from *Rac1* ^*f/f*^ and *Rac1* ^*d/d*^ uteri were analyzed for VEGFA (**C, Right**) and IGFBP4 (**D, Right**) by ELISA. Data represent mean ± SEM from three separate samples and were analyzed by two-way ANOVA with Bonferroni post-test. Asterisks indicate statistically significant differences (*P < 0.05, **P < 0.01, and ***P < 0.001).

We next investigated whether Rac1 signaling in the decidual cells exerts paracrine effects on the trophoblast cells during the early stages of placenta formation. Interestingly, our microarray analysis revealed that factors involved in vesicular trafficking are altered in *Rac1*-null decidual cells (S2 Table). In particular, we observed a marked down-regulation of mRNAs corresponding to *Rab27b*, a member of the Rab27 subfamily of GTPases, which participate in membrane trafficking and thereby control protein secretion [17, 18, 36, 37]. This subfamily consists of two closely related homologs, Rab27a and Rab27b [17]. Rab27a is expressed in a wide variety of secretory cells and participates in the exocytosis of various secretory vesicles [17]. In contrast, Rab27b expression is much more restricted and presumably tightly regulated to allow the controlled release of vesicle contents in response to appropriate physiological signals [17, 18, 36]. As shown in Fig 7A, we observed downregulation of *Rab27b* mRNA, but not *Rab27a* mRNA, in uterine decidual cells of *Rac1* ^*d/d*^ mice on day 8 of gestation. Consistent with this finding, we noted a marked decline in the levels of Rab27B protein in the uterine sections of *Rac1* ^*d/d*^ mice (Fig 7B). Since the Rab27 proteins are known to control several steps in vesicular trafficking, including vesicle movement on tubulin cytoskeletal tracks, we considered the possibility that down-regulation of *Rab27b* expression in *Rac1*-null uteri might affect secretory activity of the decidual cells.

### Rac1 controls VEGF secretion by decidual cells

Since *Rac1* ^*d/d*^ uteri exhibit defects in angiogenesis, we sought to determine whether the secretion of vascular-endothelial growth factor A (VEGFA), a potent endothelial mitogen, was affected in *Rac1*-null decidual cells. To test this possibility, we employed well-established primary cultures of murine stromal cells that undergo decidualization *in vitro* [31]. Stromal cells isolated from *Rac1* ^*f/f*^ and *Rac1* ^*d/d*^ uteri were subjected to *in vitro* decidualization with estrogen and progesterone and analyzed for the expression of VEGFA. Immunocytochemical analysis revealed that in control decidual cells collected from *Rac1* ^*f/f*^ uteri, VEGFA was noticeable as diffuse staining throughout the cell, suggesting that this factor is actively trafficked through the extensive ER-Golgi-vesicular network in the cytosol prior to its secretion in the growth medium (Fig 7C, left). In contrast, the stromal cells from *Rac1* ^*d/d*^ uteri displayed intense staining and retention of VEGFA within the cell, indicating impaired secretion, presumably due to a defective vesicular transport pathway. Consistent with this observation, our study revealed significantly reduced VEGFA in the conditioned media of decidual cells collected from *Rac1*-null uteri (Fig 7C, right).

### Rac1 controls IGFBP4 secretion by decidual cells

Phenotypic analysis of pregnant *Rac1* ^*d/d*^ uteri revealed abnormal trophoblast expansion during placentation. A critical balance of embryonic insulin-like growth factors, IGF1 and IGF2, and maternal insulin-like growth factor binding proteins (IGFBPs), which curb the actions of these growth factors, has been shown to be important in the control of trophoblast proliferation and differentiation [38–40]. We, therefore, examined whether the secretion of IGFBPs is altered in decidual cells lacking *Rac1*. Two members of the IGFBP family, IGFBP1 and IGFBP4, were previously reported to be expressed in the mouse uterus during early pregnancy [41, 42]. While IGFBP1 was expressed in mouse uterine epithelial cells, IGFBP4 expression was limited to stromal cells during the decidual phase of pregnancy [41, 42]. We, therefore, determined whether secretion of IGFBP4 is affected in decidual cells lacking *Rac1*. As shown in Fig 7D, there was significant accumulation of IGFBP4 in *Rac1*-null decidual cells, indicating a defect in the secretion of this protein. This concept received further support from the observation that the level of IGFBP4 was markedly reduced in the conditioned media of decidual cells collected from *Rac1*-null uteri (Fig 7D, right). Taken together, our results indicated that Rac1 regulates secretion of IGFBP4 by decidual cells, which in turn would be expected to control IGF-induced trophoblast proliferation and differentiation during placentation.

### Rac1 regulates VEGF secretion from human endometrial stromal cells during decidualization

**Fig 8:**
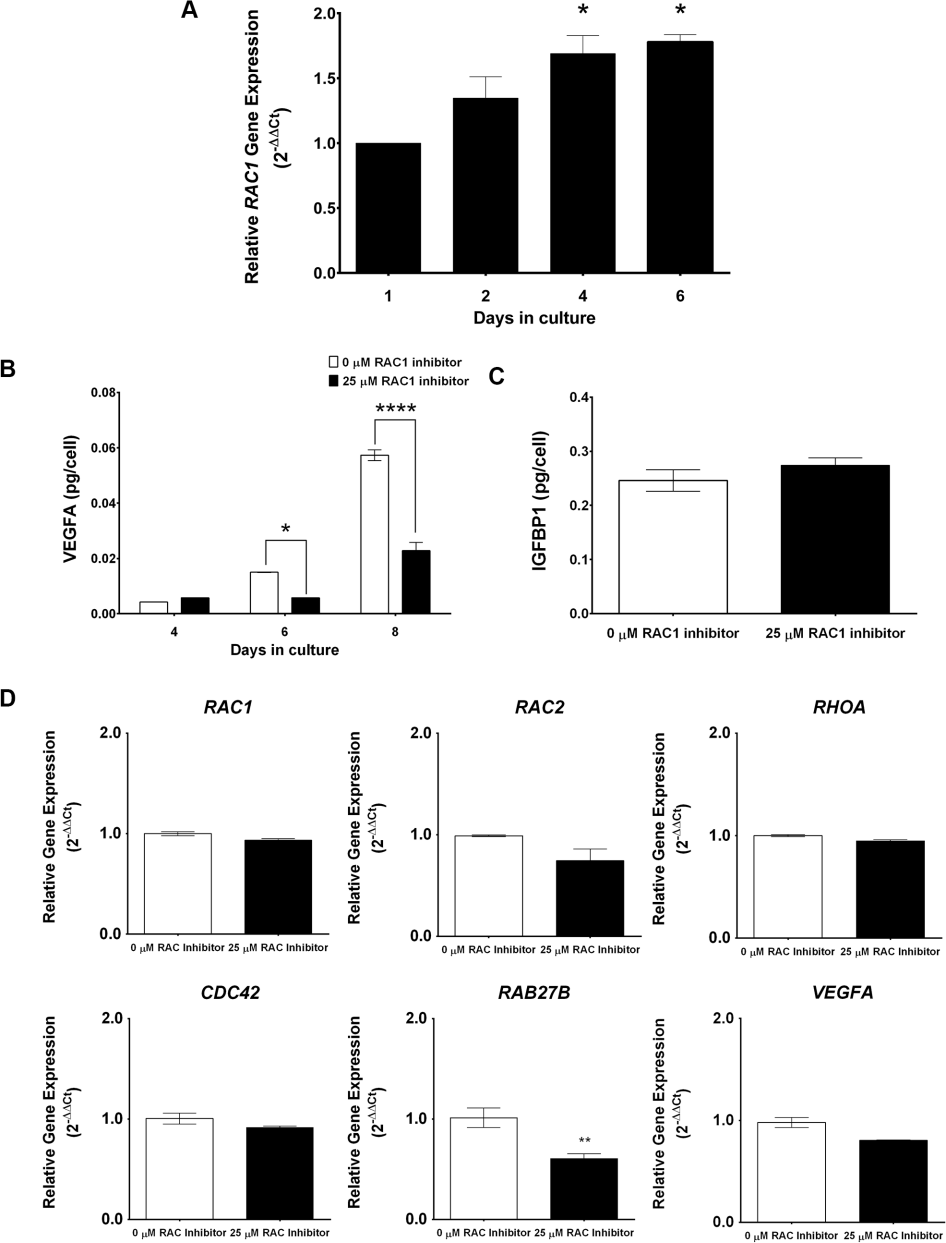
RAC1 regulates secretory function of human endometrial stromal cells. **(A)** *RAC1* is induced in primary human endometrial stromal cells undergoing *in vitro* decidualization. Human endometrial stromal cells (HESCs) were subjected to differentiation in response to estrogen, progesterone, and 8-Br-cAMP as described in the Materials and Methods. The expression of *RAC1* mRNA was assessed by qPCR. Data represent mean ± SEM from three separate samples and were analyzed by one-way ANOVA with Bonferroni post-test. Asterisks indicate statistically significant differences (*P < 0.05). **(B & C)** Inhibition of biological activity of RAC1 in HESCs inhibits *RAB27B* expression and VEGFA secretion in the conditioned media. HESCs were subjected to differentiation in the absence or presence of 25μM InSolution Rac1 Inhibitor II (Z62954982) for eight days. **(B)** qPCR was performed to monitor the expression of *RAC1*, *RAC2*, *RHOA*, *CDC42*, *VEGFA*, and *RAB27B*, N = 2–3, P< 0.001. **(C & D)** ELISA was performed to measure VEGFA and IGFBP1 secretion in the conditioned media. VEGFA ELISA data represent mean ± SEM from three separate samples and were analyzed by two-way ANOVA with Bonferroni post-test. Asterisks indicate statistically significant differences (*P < 0.05 and ***P < 0.001). IGFBP1 ELISA data represent mean ± SEM from two separate samples and were analyzed by *t*-test, *P > 0*.05.

We next assessed whether Rac1-mediated regulation of decidual secretory pathways is conserved in the human. To test this, undifferentiated human endometrial stromal cells (HESC) isolated from biopsies obtained from normal fertile women in the proliferative stage of the menstrual cycle were placed in culture and subjected to decidualization *in vitro* in response to a hormonal cocktail containing progesterone, estrogen, and 8-bromo-cAMP as described previously [43, 44]. As shown in Fig 8A, *RAC1* transcripts are induced in HESC during *in vitro* decidualization. We next employed the Rac1 inhibitor Z62954982, which specifically blocks the activation of RAC1 [45], to investigate the role of this factor in regulating the secretory pathways in differentiating HESC. Our studies revealed that treatment of endometrial stromal cells with the RAC1-specific inhibitor did not affect the expression of transcripts corresponding to *RAC1*, *RAC2*, *RHOA*, *CDC42*, or *VEGFA*, but led to marked suppression in the level of *RAB27B* transcripts (Fig 8B), indicating that the regulation of RAB27B expression by RAC1 is conserved in decidua of mouse and woman. Most importantly, inactivation of RAC1 signaling and consequent down-regulation of *RAB27B* gene expression were associated with a strong reduction in the levels of VEGFA secreted in the conditioned media of decidualizing HESC compared to untreated HESC (Fig 8C). Interestingly, while IGFBP4 levels were undetectable in the conditioned medium of decidualizing HESC, we observed significant levels of IGFBP1 in their conditioned media. The secreted IGFBP1 levels, however, remained unaffected by the presence or absence of the RAC1-inhibitor by day 8 of culture (Fig 8D). These results indicated that RAC1 regulates the secretion of VEGFA, but not that of IGFBP1, by HESC during decidualization. Collectively, our results are consistent with the hypothesis that Rac1, acting via its downstream effector Rab27b, controls the secretory pathways that operate in decidual cells to regulate the secretion of key paracrine factors, such as VEGF, in both mouse and human endometrium.

## Discussion

Rac1 is a pleiotropic factor that controls a variety of cellular events and contributes to specific differentiation processes. Its GTPase activity transduces extracellular signals from seven-transmembrane protein receptors, integrins, and growth factor receptors to effector molecules that modulate multiple signaling pathways [13–15, 46]. Activation of the mitogen-activated protein kinase pathway is a prominent mechanism that functions downstream of Rac1 in response to appropriate cellular signals [13–15, 46, 47]. Rac1 also promotes cell migration by regulating the formation of lamellopodia, which are sheet-like projections on the leading edge of a motile cell that propel it across a matrix [48, 49]. Interestingly, a previous study reported that Rac1 controls stromal cell migration during invasion of human embryo into the decidua [21]. This study was limited to *in vitro* conditions, using cell cultures, and did not address the mechanisms via which Rac1 accomplishes this function during uterine differentiation. Our study, employing *Rac1* ^*d/d*^ mice, addressed the *in vivo* function of this factor in endometrial stromal cells during decidualization and embryo invasion. Surprisingly, phenotypic analysis of *Rac1* ^*d/d*^ mice did not reveal any evidence of curtailed embryo invasion during days 5–7 of pregnancy but supported a later role of Rac1 in controlling angiogenesis and trophoblast development. Our studies showed that Rac1 plays a critical role in placenta development by regulating secretory pathways in decidual cells. However, it should be emphasized that Rac1 does not regulate all types of decidual secretions. Since the loss of uterine Rac1 does not affect pregnancy until day 7 of gestation, we infer that Rac1 is unlikely to control decidual secretions during days 5–7 of gestation. Based on our results, we postulate that Rac1 acts within a critical time window that overlaps with days 8–10 of mouse pregnancy, to guide specific cellular mechanisms that regulate the decidual secretion of certain key factors, VEGFA and IGFBP4, which influence the activity of endothelial and trophoblast cells, respectively. Mice lacking *Rac1* in the decidua, therefore, present a unique model in which one can study the mechanisms by which maternal endometrial secretory pathways regulate angiogenesis and trophoblast development.

A major phenotypic consequence of the loss of Rac1 signaling in the decidua is a drastic decrease in the development of the uterine vascular network that supports embryonic growth. We found that Rac1 controls angiogenesis by regulating the expression of several factors with known roles in this complex process, including neuropilin 1 (Nrp1), angiopoietin 2 (Angpt2), and sphingosine kinase 1 (Sphk1). Nrp1, a co-receptor for VEGFA and semaphorin family members, has widespread functions in angiogenesis, axonal guidance, cell survival, migration, and invasion [50]. Angpt2, another key regulator of angiogenesis, binds the endothelial-specific receptor tyrosine kinase 2 (TIE2) to control sprouting of blood vessels. In a context-dependent manner, it can either act on TIE2-positive endothelial cells to antagonize the action of angiopoietin-1 or exert a proangiogenic effect on less mature TIE2-negative endothelial cells [51]. Sphk1, which controls sphingolipid signaling, prevents vascular leakage during uterine angiogenesis [52]. It is conceivable that the downregulation of Sphk1 contributes to the observed vascular leakiness and hemorrhage in *Rac1* ^*d/d*^ uteri. Interestingly, Rac1 does not regulate the expression of VEGFA by decidual cells but controls its secretion. Very little is known about the mechanisms via which cells release VEGF to their surroundings, but different isoforms are known to be differentially soluble. Using green fluorescent protein-tagged VEGF, it was reported that the VEGF enters the early ER-Golgi secretory steps, but its secretion may involve trafficking mechanisms distinct from the “constitutive” secretory pathway [53, 54]. Interestingly, a substantial fraction of VEGF-GFP is released from the cell surface by shedding, possibly as cargo contained inside extracellular vesicles [53, 54]. This raised the possibility that Rac1 controls vesicular trafficking to regulate the secretory activity of decidual cells.

Consistent with this prediction, gene expression profiling revealed that Rac1 regulates the expression of Rab27b, a member of the Rab subfamily of GTPases with restricted expression that regulates exocytic pathways of various secretory vesicles [17, 18, 36, 37]. Such mechanisms allow the controlled release of dense-core granules or secretory granules, only in response to appropriate physiological signals [55]. Rab27b is known to play a critical role in regulated stimulus-induced (*vs*. constitutive) exocytosis and has been shown to control secretion of platelet dense granules and pancreatic acinar granules [18, 36]. Since decidual cells possess secretory granules [56], it is conceivable that Rac1-Rab27b pathway mediates their regulated exocytosis of secretory granules to control endometrial function in a stage-specific manner. Further investigation is needed to clarify the role of this signaling factor in controlling decidual cell secretions.

**Fig 9:**
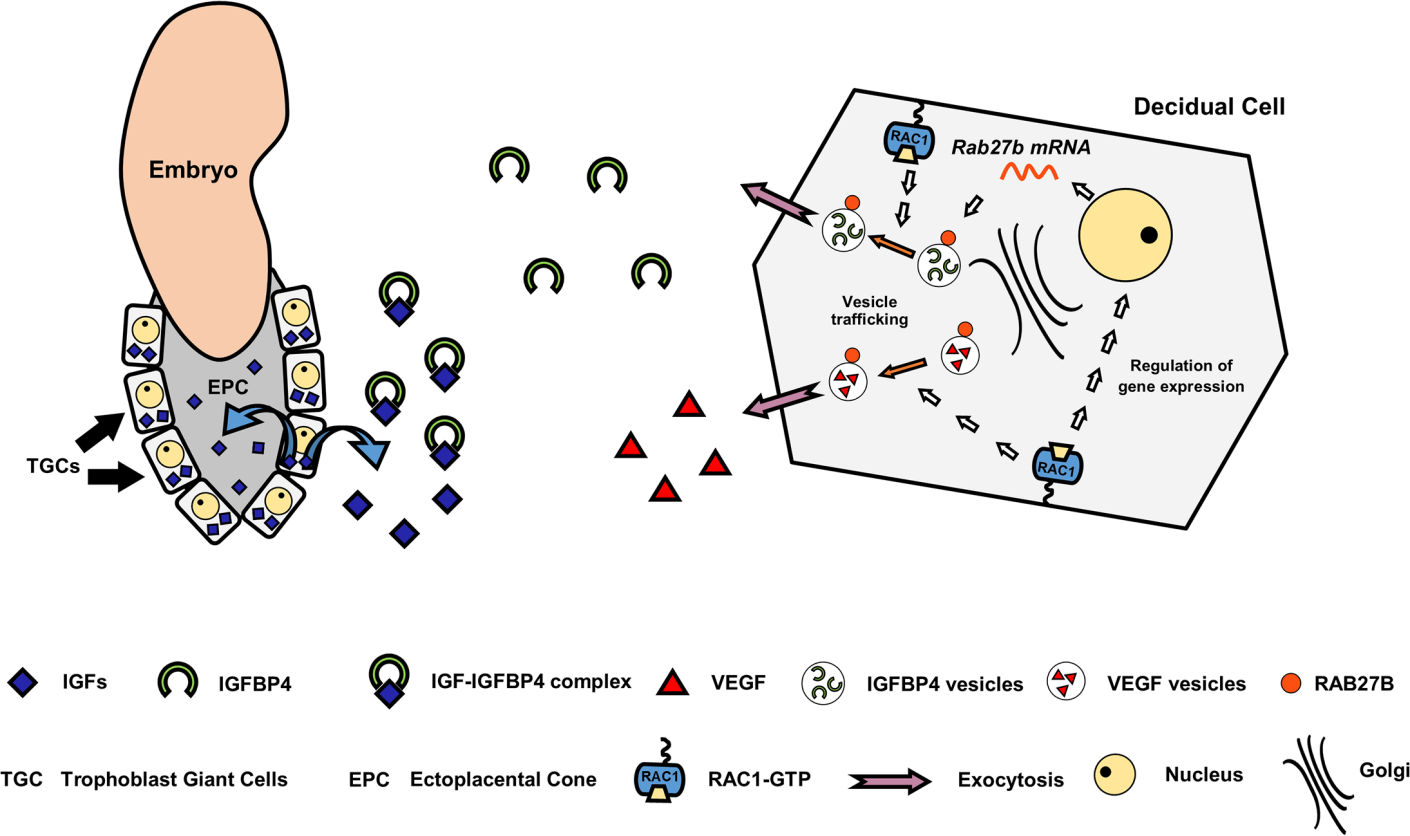
A model depicting the mechanism of Rac1 action in mouse uterus during early pregnancy.

Another major finding of this study is that decidual Rac1 regulates development of the placenta, presumably by controlling the secretion of IGFBP4. Following implantation of the blastocyst into the uterus, trophoblastic cells in the EPC of mouse embryos must proliferate and differentiate into TGCs. It was previously shown that IGF1 promotes the proliferation of EPC cells while IGF2 induces their transformation into TGCs, which invade into the uterine tissue to gain access to the maternal blood supply. It is generally thought that autocrine secretion of IGFs by the embryo drive trophoblast proliferation and migration, whereas maternal decidua modulates their actions by secreting IGFBPs, which control IGF bioavailability. The expression of four types of IGFBPs, IGFBP1, IGFBP2, IGFBP3, and IGFBP4 by human decidual cells has been reported [57]. In mice, while IGFBP-1 is detected in uterine epithelial cells, IGFBP-4 is the predominant IGFBP in stromal cells during the decidual phase of pregnancy [41, 42]. Indeed, [^125^I]IGF1 ligand blot analysis of mouse uterine tissue extracts showed that only IGFBP4 was significantly increased during early pregnancy [41, 42]. It is tempting to suggest that IGFBP4, which is known to bind both IGF1 and IGF2, regulates trophoblast proliferation and differentiation in the EPC by buffering the bioavailable IGFs. The *Rac1* ^*d/d*^ model, therefore, provides a plausible link between impaired secretion of a critical decidual IGFBP and the observed TGC defect in placenta development. A model depicting the role of RAC1 in endometrial angiogenesis and placental development is shown in Fig 9. Interestingly, Nagashima *et al* recently reported that conditional deletion of bone morphogenetic protein receptor type 2 (BMPR2) in the uterine decidua leads to abnormal vascular development, expansion of TGCs, and a deficiency of uterine natural killer cells [58]. Disruption of these pathways collectively impairs placental function and promotes fetal demise by midgestation in *Bmpr2* conditional knockout mice. While there are phenotypic similarities between the *Rac1* ^*d/d*^ and *Bmpr* ^*d/d*^ mouse models, it remains to be determined whether RAC1 and BMPR2 pathways converge or they function via distinct mechanisms.

It is of interest that Rac1 regulation of the decidual cell secretory pathways, particularly that of VEGFA, is conserved in the mouse and the human. While the secreted IGFBP1 levels remained unaffected by the presence or absence of the RAC1-inhibitor in human endometrial stromal cells during the first 6–8 days of culture, we cannot rule out the possibility that RAC1-mediated regulation of IGFBP1 requires a longer duration of culture. Alternatively, it is possible that the human *in vitro* endometrial culture system does not fully capture the decidual program that occurs *in vivo*. Nonetheless our finding with the human endometrial stromal cultures has important clinical implications. Human placental insufficiency syndromes, including intrauterine growth restriction (IUGR) and preeclampsia, are characterized by abnormalities in blood vessel network formation, and trophoblast differentiation and function [59–61]. In pregnancies with IUGR, decreased expression of VEGFA and aberrant expression of angiopoietins are associated with poor placental blood vessel development [62, 63]. Reduced expression of VEGFA has also been reported in endometrium of women with recurrent miscarriage [64]. Other studies have shown that dysregulated IGFBP1 or IGFBP4 is associated with IUGR [65, 66]. Collectively, these results indicate that inappropriate Rac1 signaling in human decidua may result in impaired angiogenesis, vascular disruption, hemorrhage and aberrant trophoblast proliferation and differentiation, resulting in pregnancy loss. Further studies using human endometrial specimens obtained from patients with recurrent pregnancy loss may reveal whether aberrant RAC1 signaling is linked to dysregulated endometrial secretions, contributing to disruption of the maternal-embryo coordination, affecting placentation, and causing miscarriage.

## Materials and Methods

### Ethics statement

Mice were maintained in the animal facility at the University of Illinois, College of Veterinary Medicine, in accordance to the institutional guidelines for the care and use of laboratory animals and in accordance with the National Institutes of Health standards for the use and care of animals. The Institutional Animal Use and Care Committee at the University of Illinois at Urbana-Champaign approved all procedures involving animal care, euthanasia, and tissue collection.

### Animals

Mice were housed in an animal room with temperature of 22°C and 12L:12D cycles. Food and water were provided ad libitum. Mice harboring a ‘floxed’ *Rac1* gene (*Rac1* ^*tm1Glog/ tm1Glog*^ */J*, Jackson Laboratory) were mated with mice that express Cre recombinase under the control of the progesterone receptor promoter (*Pgr* ^*tm2(cre)Lyd/+*^), termed *Prg* ^*Cre/+*^. The *Pgr-Cre* mice were kindly provided by Drs. Francesco J. DeMayo and John P. Lydon of Baylor College of Medicine. The crossing of the above transgenic animals was used to produce the founding colony that produced the experimental mice containing the following genotypes: *Pgr* ^*+/+*^ *Rac1* ^*f/f*^ (termed *Rac1* ^*f/f*^), *Pgr* ^*Cre/+*^ and *Rac1* ^*f/f*^ (termed *Rac1* ^*d/d*^). This strategy has been used extensively to ablate ‘floxed’ genes in tissues expressing PGR [20, 24–27].

### Chemicals, reagents, and antibodies

Progesterone (P_4_), 17β-estradiol (E_2_), naphthol AS-MX phosphate, Fast Blue RR (4-benzoylamino-2,5-dimethoxyaniline diazonium), collagenase, pancreatin, dimethyl sulfoxide (DMSO), 8-bromoadenosine 3',5'-cyclic monophosphate salt (cAMP), and Trypan blue were purchased from Sigma. Hanks Balanced Salt Solution (HBSS), dispase, Dulbecco’s modified Eagle medium-F12 medium HEPES, no phenol red (DMEM/F12), Penicillin-Streptomycin, Fungizone, and Phalloidin conjugated to Alexa 488 were purchased from Life Technologies. Fetal bovine serum (FBS) was purchased from Fisher Scientific. InSolution Rac1 Inhibitor II (Z62954982) was purchased from Millipore. Fluoromount-G with DAPI was purchased from eBiosciences.

Uterine sections or endometrial stromal cells were incubated with one or more of the following primary antibodies: activated RAC1 (RAC1-GTP, 1:50, NewEast Biosciences, 26903), proliferating cell nuclear antigen (PCNA, 1:200, Santa Cruz Biotechnology, SC-56), platelet/endothelial cell adhesion molecule 1 (PECAM1/CD31, 1:500, BD Pharmigen, 557355), decidual prolactin-related protein (PRL8A2/dPRP, 1:1000, a generous gift from Dr. Michael Soares, University of Kansas), vascular endothelial growth factor A (VEGFA, 1:100, Santa Cruz Biotechnology, SC-152), cytokeratin 8 (KRT8, 1:50, Developmental Studies Hybridoma Bank, TROMA-I), β-tubulin (TUBB, 1:50, Developmental Studies Hybridoma Bank, E7), insulin-like growth factor-binding protein 4 (IGFPB4, 1:200, Novus, NBP1-80549), placental lactogen 1 (PL1, 1:200, Santa Cruz Biotechnology, SC-34713), Ras-related in brain 27B (RAB27B, 1:200, Santa Cruz Biotechnology, SC-22993), and trophoblast specific protein alpha (TPBPA, 1:200, Abcam, ab104401).

The fluorescent-tagged secondary antibodies and normal donkey serum were purchased from Jackson ImmunoResearch. The following secondary antibodies were used: rhodamine or Cy3 donkey anti-rabbit, 488 donkey anti-rabbit, 488 donkey anti-mouse, 488 donkey anti-goat, and Cy3 donkey anti-rat. For immunocytochemistry, F-actin filaments were stained using phalloidin conjugated to Alexa 488.

### Fertility assessments, timed pregnancies, and tissue collection

To test fertility, *Rac1* ^*f/f*^ and *Rac1* ^*d/d*^ mice of reproductive age (7–8 weeks) were paired with fertile wild-type males for six months. The total number of pups born in each litter and the number of pregnancies during this period was recorded.

For experiments involving timed pregnancies, female mice were mated with adult wild-type males of known fertility. For tissue collection, all animals were euthanized by CO_2_ asphyxiation. Uteri and ovaries were collected at different time points during pregnancy and the tissues were immersion-fixed in 10% (vol/vol) neutral-buffered formalin (NBF) for histological evaluation or flash frozen in liquid N_2_ for RNA isolation or frozen sectioning. As a reference for our experiments, the identification of a copulatory plug indicated day 1 of pregnancy.

### Serum hormone assay

Following euthanasia, blood was drawn via cardiac puncture using a 30 gauge needle and transferred into a sterile 1.5 mL tube. The blood samples were incubated at room temperature for 90 min to allow clot formation. After the incubation, the clot was removed with a sterile pipette tip and the samples were spun at 2000 x *g* for 15 min at room temperature. The serum samples were transferred into a new sterile 1.5 mL tube and stored at -80°C until analyzed. Serum hormones were measured by radioimmunoassay at the Ligand Core facility, University of Virginia, Charlottesville.

### Experimentally-induced decidualization

Uterine stromal cell decidualization was experimentally induced in adult non-pregnant, hormone-primed mice as described previously [20]. Briefly, *Rac1* ^*f/f*^ and *Rac1* ^*d/d*^ female mice were ovariectomized to remove any circulating hormones. Two weeks following ovariectomy, animals were injected with 100 ng of E_2_ in 0.1 mL of corn oil subcutaneously (sc) every 24 h for three consecutive days. After two days of rest, sc hormones injections were given daily, containing 1 mg P_4_ and 10 ng E_2_ in 0.1 mL for three consecutive days. Decidualization was initiated in one horn by injecting 20 μL of oil into the lumen, while the other horn was left unstimulated. Mice were treated with additional E_2_ + P_4_ for up to 96 h post-stimulus. Mice were euthanized, uterine horns were collected and weighed.

### Alkaline phosphatase activity

Alkaline phosphatase (ALPL) activity was detected following previously published protocols [67], with modifications. Briefly, frozen uterine sections were fixed in 10% NBF for 10 min, and then washed with 1x phosphate-buffered saline (PBS) three times for 5 min each. The uterine sections were then incubated in the dark at 37°C for 30 min in a solution containing 0.5 mM naphthol AS-MX phosphate (ALPL substrate) and 1.5 mM Fast Blue RR in 0.1 M Tris-HCl, pH 8.5. Alkaline phosphatase activity releases orthophosphate and naphthol derivatives from the ALPL substrate. The naphthol derivatives are simultaneously coupled with the diazonium salt (Fast Blue RR) to form a dark dye marking the site of enzyme action. The slides were rinsed in tap water to terminate the enzymatic reaction. Stained uterine sections were visualized under an Olympus BX51 microscope equipped for light imaging and connected to a Jenoptik ProgRes C14 digital camera with c-mount interface containing a 1.4 Megapixel CCD sensor.

### Primary mouse endometrial stromal cell isolation and induction of decidualization

Mouse endometrial stromal cells (MESC) were isolated from uteri on day 4 pregnancy, as previously described [31]. Briefly, uteri collected from *Rac1* ^*f/f*^ and *Rac1* ^*d/d*^ female mice were cut open and digested with 5 mL/uteri with 1x HBSS solution containing 6 g/L dispase and 25 g/L pancreatin for 45min at room temperature, followed by 15 min at 37°C, with occasional mixing. After the first digestion, the supernatant, which contains epithelial cells, was removed by aspiration and the remaining pieces of tissues were washed with HBSS containing 10% (vol/vol) heat-inactivated fetal bovine serum (FBS) to stop the enzymatic digestion. The uterine fragments were washed two times with 1x HBSS to remove the serum. After the last wash, the uterine fragments were digested in 5 mL/uteri with 1x HBSS solution containing 0.5 g/L collagenase for 1 h at 37°C. After the second digestion, 5 mL of HBSS containing 10% FBS was added to stop the enzymatic digestion. The tubes were vortexed for 10–15 seconds until the supernatant became turbid with dispersed cells. The content of the tube is passed through an 80-μm gauze filter (Millipore) into a new collection tube to remove the undigested myometrial fragments. The suspension containing the endometrial cells was then spun at 430 x *g* for 5 min to form a pellet, and the pellet is washed with HBSS once. After the wash, the endometrial cells are spun again and resuspended in DMEM/F12 supplemented with 2% FBS, 100 units/L Penicillin, 0.1 g/L Streptomycin, 1.25 mg/L Fungizone, 10 nM E_2_, and 1 μM P_4_. The numbers of live cells were assessed by trypan blue staining using a hemocytometer. Mouse endometrial stromal cells were seeded in 6-well plates at an initial plating density of 5 × 10^5^ cells. The unattached cells were removed by washing several times with HBSS, and cell culture was continued after addition of fresh culture medium. Culture medium was collected at 72 h and 96 h after the initial plating and stored at -80°C until assayed. At the end of the culture, the cells were detached from the plates, counted using Trypan Blue and a hemocytometer, and stored at -80°C for RNA extraction.

### *In vitro* decidualization of HESC

The studies involving human endometrial stromal cell (HESC) cultures adhere to the regulations set forth for the protection of human subjects participating in clinical research and are approved by the Institutional Review Boards of Emory University, Wake Forest University, and the University of Illinois at Urbana-Champaign. Endometrial samples from the early proliferative stage of the menstrual cycle were obtained by Pipelle biopsy from regularly cycling, fertile volunteers on no hormonal medications, after providing written informed consent. HESC were maintained in a media containing DMEM/F-12 supplemented with 5% FBS, 100 units/L Penicillin, 0.1 g/L Streptomycin, as described previously [31, 44]. The culture medium was changed every 48 h.

Human endometrial cells were seeded in 6-well plates at an initial plating density of 2 × 10^5^ cells, and cultured to 90% confluence. To inhibit the activation of RAC1 in HESCs, the cells were cultured in media containing 25 μM of RAC1 inhibitor, which inhibits RAC1-TIAM1 interaction [45] or DMSO control for 24 h. To induce *in vitro* decidualization, the cells were treated with medium containing a hormonal cocktail consisting of 10 nM E_2_, 1 μM P_4_, 0.5 mM cAMP and 25 μM of RAC1 inhibitor (IC50 = 12.2 μM) or DMSO control. The culture medium containing the inhibitor or DMSO, was changed every 48 h and the cultures were maintained for up to 8 days. Conditioned media were collected on days 6 and 8 of culture and stored at -80°C until assayed. At the end of the culture, the cells were detached from the plates, counted, and stored at -80°C for RNA extraction.

### Immunohistochemistry (IHC) and immunocytochemistry (ICC)

Paraffin-embedded and/or frozen uterine sections were subjected to immunohistochemistry as described previously [20]. Briefly, tissues were collected and fixed in 10% NBF for 18–24 h or flash frozen in liquid nitrogen. Fixed tissues were embedded in paraffin, sectioned at 5 μm, mounted on glass slides, and incubated at 37°C overnight. Tissue sections were deparaffinized in xylene, rehydrated through a graded series of ethanol, and washed in tap water. For most of the immunostaining, antigen retrieval was performed in a pressure cooker in 10 mM sodium citrate buffer (pH 6.0) for 20 min and then the slides were cooled to room temperature. For RAC1-GTP, specifically, antigen retrieval was performed by incubating the slides in 10 mM sodium citrate buffer (pH 6.0) for 2 h at 80°C in a water bath. When frozen tissues were used, the sections were thawed at room temperature for 5 min and then fixed for 5 min in 10% NBF. For both paraffin-embedded and frozen tissues, washes between steps (three times for 5 min each) were done using 1x phosphate-buffered saline solution containing 0.05% Tween 20 (PBS-T). Nonspecific binding was inhibited by incubating the sections with 10% normal serum for 1 h at room temperature. After the serum block, sections were incubated overnight at 4°C with the diluted antibody solution in PBS-T containing 1% normal serum. Labeling was visualized by incubation with a fluorescent-tagged secondary antibody for 1 h at room temperature. All incubations were done using a humidified chamber protected from light. Slides were mounted using a mounting solution containing DAPI. Pictures were taken using the Olympus BX51 microscope equipped for fluorescent imaging and connected to a Jenoptik ProgRes C14 digital camera with c-mount interface containing a 1.4 Megapixel CCD sensor. Fluorescent images were processed and merged using Adobe Photoshop Extended CS6 (Adobe Systems).

Primary cultures of stromal cells were subjected to immunocytochemistry as described previously [31]. Briefly, cells were fixed in 10% NBF for 10 min, and then washed with PBS. Cells were then permeabilized using PBS containing 0.1% Triton X for 10 min at room temperature. Nonspecific binding was inhibited by incubating the sections with 10% normal serum for 1 h at room temperature. After the serum block, the cells were incubated overnight at 4°C with the diluted antibody solution in PBS containing 1% normal serum. Labeling was visualized by incubation with a fluorescent-tagged secondary antibody for 1 h at room temperature. One drop of mounting solution containing DAPI was added to each well to stain the nucleus. Pictures were taken using the Olympus Ix70 inverted microscope adapted to a Diagnostic Instrument digital camera containing a 2.0 Megapixel CCD sensor. Fluorescent images were merged and processed using Adobe Photoshop Extended CS6.

### DNA microarray sample collection and analysis

Decidualization was experimentally induced in adult non-pregnant, hormone-primed mice as described previously [20]. Briefly, mice were ovariectomized to remove any circulating hormones. Two weeks following ovariectomy, animals were injected with 100 ng of E_2_ in 0.1 mL of corn oil sc every 24 h for three consecutive days. After two days of rest, sc hormones injections were given daily, containing 1 mg P_4_ and 10 ng E_2_ in 0.1 mL for three consecutive days. Decidualization was initiated in one horn by injecting 20 μL of oil into the lumen, while the other horn was left unstimulated. Mice were treated with additional E_2_ + P_4_ for up to 72 h post-stimulus. Mice were euthanized, uterine horns were collected and weighed. Total RNA was extracted from stimulated and unstimulated uterine horns using a standard TRIzol-based protocol. RNA integrity was verified using Agilent 2100 bioanalyser (Agilent Technologies Inc., Santa Clara, CA, USA) at the Biotechnology Center of the University of Illinois, Urbana and Champaign. Each RNA sample was processed for microarray hybridization using Affymetrix GeneChip Mouse Genome 430A 2.0 array, which contains probes that represented approximately 14,000 annotated gene sequences, following the established protocol. A list of genes that had a relative fold change of >1.3 were further sorted by gene ontology and pathway analysis using Ingenuity Classification Software.

To investigate the biological pathways affected by *Rac1* deletion in the uterus, we performed gene expression profiling using uteri from *Rac1* ^*f/f*^ and *Rac1* ^*d/d*^ mice. Briefly, uterine decidual masses were isolated and freed from the embryo from day 8 pregnant *Rac1* ^*f/f*^ and *Rac1* ^*d/d*^ mice. Because *Rac1* ^*d/d*^ mice exhibit hemorrhage at this time of pregnancy, all animals were perfused with 1x HBSS containing 200 U of heparin/mL. Total RNA was extracted from the decidual masses using a standard TRIzol-based protocol. The RNA preparations were then purified with the RNeasy kit (Qiagen). The purity and quality of the isolated RNA samples were assessed using an Agilent Bioanalyzer System and samples with a RNA integrity number of 10 were used. The RNA was hybridized to the Affymetrix GeneChip Mouse Genome 430A 2.0 array, which contains probes that represented approximately 14,000 annotated gene sequences. RNA quality and chip hybridization was performed by Roy J. Carver Biotechnology Center at the University of Illinois of Urbana-Champaign. Subsequently, the chips were scanned and the data were extracted using Gene-Chip Operating Software version 1.3 (Affymetrix). A signal value for each gene below 50 was considered as background. The relative gene expression fold value was determined by the ratio of gene expression of *Rac1* ^*d/d*^ decidua to *Rac1* ^*f/f*^ decidua. A list of genes that had a relative fold change of > 1.3, were further analyzed for gene ontology and functional classification using the Database for Annotation, Visualization and Integrated Discovery (DAVID, National Institute of Allergy and Infectious Diseases, National Institutes of Health)[68].

### Quantitative real time PCR analysis (qPCR)

Total RNA was isolated from uteri, ovaries, and cells using a standard TRIzol-based protocol. The RNA concentration of each sample was determined at 260 nm using a Nanodrop ND1000 UV-Vis spectrophotometer (Nanodrop Technologies). RNA samples were reverse transcribed using the High Capacity cDNA Reverse Transcription kit (Applied Biosystems) according to the manufacturer's instructions. Primers specific for genes of interest were developed and real-time quantitative PCR (qPCR) reactions were carried out using SYBR-green master mix (Applied Biosystems) in a 7500 Applied Biosystems Real-time PCR machine (Applied Biosystems). For each sample, the mean threshold cycle (Ct) was calculated from Ct values obtained from three replicates. The normalized ΔCt in each sample was calculated as mean Ct of target gene subtracted by the mean Ct of the reference gene. ΔΔCt was then calculated as the difference between the ΔCt values of the control and mutant samples. The fold change of gene expression in each sample relative to a control was generated using the 2^−ΔΔCt^ mathematical model for relative quantification of quantitative PCR [69]. The mean fold induction and SEM were calculated from at least three or more independent experiments. For HESC, the mean fold induction and SEM were calculated from at least two independent experiments. The housekeeping gene *RPLP0* (*36B4*), which encodes a ribosomal protein, was used as a reference gene. Reported data consists of mean fold induction ± SEM.

### Measurement of secreted proteins by ELISA

The media of MESCs and HESCs were collected at different time points, as described above, from at least 3 wells of a 6-well plate and were stored at -80°C. Media samples were subjected to enzyme-linked immunosorbent assays (ELISA) and the data were analyzed according to the manufacturer's instructions. All samples were measured in duplicates and the total protein content of the culture medium was calculated. Some samples were diluted to match the dynamic range of each ELISA kit. Mean protein production was normalized to the cell counts of each sample assayed. Protein estimates were obtained from at least two independent samples for HESC and at least three independent samples for MESC. The data are reported as mean protein per cell ± S.E.M.

The analytical sensitivities of each kit were: 3 pg/mL for the mouse VEGF ELISA kit (MMV00, R&D Systems), 6.4 pg/mL for the mouse IGFBP4 ELISA kit (SEA055Mu, Uscn Life Science Inc.), 9 pg/mL for the human VEGF ELISA kit (DDV00, R&D Systems), < 5 pg/mL for the human IGFBP1 ELISA kit (ab100539, Abcam).

### Statistical analyses

Experimental data were collected from a minimum of four independent samples, which were subjected to the same experimental conditions. All numerical data are expressed as mean ± SEM. Statistical analysis was done using one of the following: Student’s *t-*test or Mann-Whitney rank sum test (for single comparison), one-way analysis of variance (ANOVA) with a Bonferroni post-test (for multiple comparison between samples or time points), or two-way ANOVA with a Bonferroni post-test (for multiple comparison between different samples and time points). Analysis of equal variances was done on all numerical data to determine if a parametric or non-parametric hypothesis testing was appropriate. Data were considered statistically significant at *P* < 0.05. All data were analyzed and plotted using GraphPad Prism 6.0 (GraphPad Software).

## Supporting Information

S1 Fig Ovarian functions and preimplantation events remain unaffected in *Rac1* ^*d/d*^ mice. **(A)** H and E staining of ovarian sections from *Rac1* ^*f/f*^ and *Rac1* ^*d/d*^ mice on days 4, 8, and 12 of pregnancy. CL indicate corpora lutea. **(B) Left:** Pre-implantation embryos were recovered from uteri of *Rac1* ^*f/f*^ and *Rac1* ^*d/d*^ mice in the morning of day 4 of pregnancy and counted. **Right:** Photograph showing embryos recovered from uteri of *Rac1* ^*f/f*^ and *Rac1* ^*d/d*^ mice. Data represent mean ± SEM from twelve separate samples and were analyzed by two-way ANOVA with Bonferroni post-test, *P > 0*.05. **(C)** Progesterone levels in serum of *Rac1* ^*f/f*^ and *Rac1* ^*d/d*^ mice on days 4, 8, 10 and 12 of pregnancy. Data represent mean ± SEM from three or four separate samples and were analyzed by two-way ANOVA with Bonferroni post-test, *P > 0*.05.
(TIF)

S2 Fig (A) Expression of factors related to angiogenesis in *Rac1* ^*d/d*^ uteri. qPCR was performed to monitor the expression of *Hif1α*, *Egln1*, *Flt1*, *Flk1*, and *Tie2* receptors in the uteri of *Rac*1^*f/f*^ and *Rac*1^*d/d*^ mice on day 8 of pregnancy. Data represent mean ± SEM from two separate samples and were analyzed by *t*-test, *P > 0*.05. **(B) Hemorrhage in *Rac1*** ^***d/d***^ **uteri.** Eosin-Y staining of uterine sections from *Rac1* ^*f/f*^ and *Rac1* ^*d/d*^ mice on day 8 of pregnancy. Panels c and d indicate magnified images of boxed area in panels a and b, respectively, and show decidual blood extravasation in *Rac*1^*d/d*^ mice.
(TIF)

S1 Table Altered expression of factors related to angiogenesis in *Rac1* ^*d/d*^ uteri. (DOCX)

S2 Table Altered expression of factors related to vesicular trafficking in *Rac1* ^*d/d*^ uteri. (DOCX)
