# Supplementary figures and images for: Rac1 Regulates Endometrial Secretory Function to Control Placental Development
Source: PLoS Genet. 2015 Aug 25;11(8):e1005458. doi: 10.1371/journal.pgen.1005458 (PMC4549291; doi:10.1371/journal.pgen.1005458)

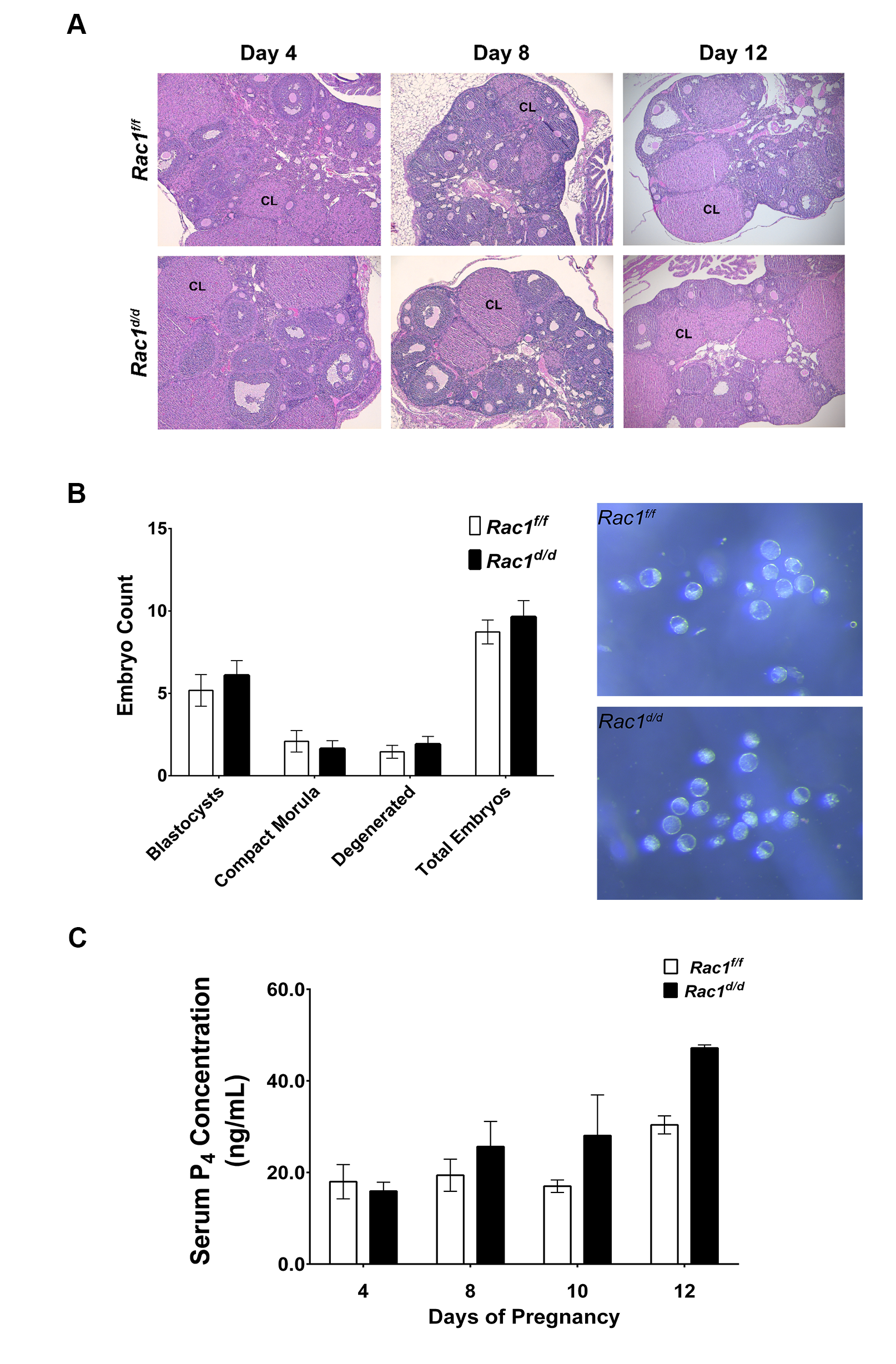

Supplement: S1 Fig — (A) H and E staining of ovarian sections from Rac1 f/f and Rac1 d/d mice on days 4, 8, and 12 of pregnancy. CL indicate corpora lutea. (B) Left: Pre-implantation embryos were recovered from uteri of Rac1 f/f and Rac1 d/d mice in the morning of day 4 of pregnancy and counted. Right: Photograph showing embryos recovered from uteri of Rac1 f/f and Rac1 d/d mice. Data represent mean ± SEM from twelve separate samples and were analyzed by two-way ANOVA with Bonferroni post-test, P > 0.05. (C) Progesterone levels in serum of Rac1 f/f and Rac1 d/d mice on days 4, 8, 10 and 12 of pregnancy. Data represent mean ± SEM from three or four separate samples and were analyzed by two-way ANOVA with Bonferroni post-test, P > 0.05. (TIF) [file pgen.1005458.s001.tif]

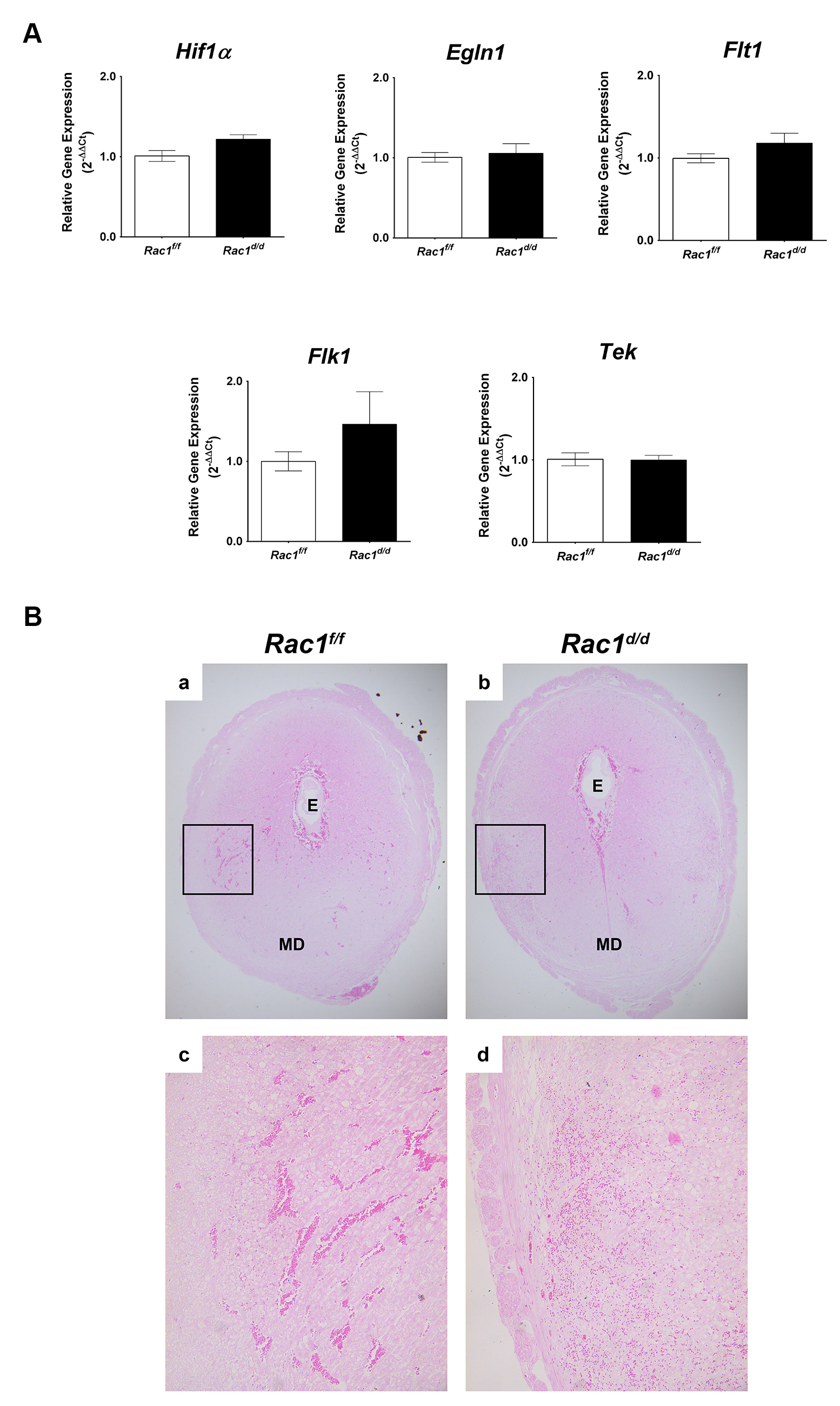

Supplement: S2 Fig — qPCR was performed to monitor the expression of Hif1α, Egln1, Flt1, Flk1, and Tie2 receptors in the uteri of Rac1f/f and Rac1d/d mice on day 8 of pregnancy. Data represent mean ± SEM from two separate samples and were analyzed by t-test, P > 0.05. (B) Hemorrhage in Rac1 d/d uteri. Eosin-Y staining of uterine sections from Rac1 f/f and Rac1 d/d mice on day 8 of pregnancy. Panels c and d indicate magnified images of boxed area in panels a and b, respectively, and show decidual blood extravasation in Rac1d/d mice. (TIF) [file pgen.1005458.s002.tif]
