# Supplementary material for: Rac1 Regulates Endometrial Secretory Function to Control Placental Development
Source: PLoS Genet. 2015 Aug 25;11(8):e1005458. doi: 10.1371/journal.pgen.1005458 (PMC4549291; doi:10.1371/journal.pgen.1005458)
Supplement: S1 Table — (DOCX) [file pgen.1005458.s003.docx]

**Table S1. Altered expression of factors related to angiogenesis in *Rac1^d/d^* uteri**

| Biological  Process | Unigene | Gene  Symbol | Full  Name | Fold Change  (*Rac1^d/d^* vs. *Rac1^f/f^*) |
| --- | --- | --- | --- | --- |
| Angiogenesis and Vascular Stability |  |  |  |  |
|  | Mm.4791 | *Ereg* | Epiregulin | 2.89 |
|  | --- | *Ang* | Angiogenin, RNase A family, 5 | 2.04 |
|  | Mm.29564 | *Mmp2* | Matrix metallopeptidase 2 | 1.74 |
|  | Mm.250981 | *Ephb2* | Eph receptor B2 | 1.67 |
|  | Mm.100068 | *Amot* | Angiomotin | 1.44 |
|  | Mm.3085 | *Plxnd1* | Plexin D1 | 1.35 |
|  | Mm.234965 | *Foxp1* | Forkhead box P1 | – 1.31 |
|  | Mm.1415 | *Epas1* | Endothelial PAS domain protein 1 | – 1.32 |
|  | Mm.21767 | *Cdh5* | Cadherin 5 | – 1.42 |
|  | Mm.356578 | *Meis1* | Meis homeobox 1 | – 1.45 |
|  | Mm.439874 | *Angpt2* | Angiopoietin 2 | – 1.78 |
|  | Mm.189536 | *Angpt4* | Angiopoietin 4 | – 1.98 |
|  | Mm.4406 | *Mmp9* | Matrix metallopeptidase 9 | – 2.01 |
|  | Mm.271745 | *Nrp1* | Neuropilin 1 | – 2.06 |
|  | Mm.275434 | *Ptgs1* | Prostaglandin-endoperoxide synthase 1 | – 2.60 |
|  | Mm.292547 | *Ptgs2* | Prostaglandin-endoperoxide synthase 1 | – 2.93 |
|  | Mm.20944 | *Sphk1* | Sphingosine kinase 1 | – 3.10 |
|  | Mm.235105 | *F13a1* | Coagulation factor XIII, A1 subunit | – 10.34 |
