# Supplementary material for: Rac1 Regulates Endometrial Secretory Function to Control Placental Development
Source: PLoS Genet. 2015 Aug 25;11(8):e1005458. doi: 10.1371/journal.pgen.1005458 (PMC4549291; doi:10.1371/journal.pgen.1005458)
Supplement: S2 Table — (DOCX) [file pgen.1005458.s004.docx]

**Table S2. Altered expression of factors related to vesicular trafficking in *Rac1^d/d^* uteri**

| Biological  Process | Unigene | Gene  Symbol | Full  Name | Fold Change  (*Rac1^d/d^* vs. *Rac1^f/f^*) |
| --- | --- | --- | --- | --- |
| Vesicular trafficking |  |  |  |  |
|  | Mm.479655 | *Syt4* | Synaptotagmin IV | 1.71 |
|  | Mm.271744 | *Pfn2* | Profilin 2 | 1.57 |
|  | Mm.35814 | *Il11* | Interleukin 11 | 1.54 |
|  | Mm.6225 | *Stx1a* | Syntaxin 1A | 1.49 |
|  | Mm.28650 | *Rab6* | RAB6 | 1.36 |
|  | Mm.298798 | *Cdk5* | Cyclin-dependent kinase 5 | 1.35 |
|  | Mm.10699 | *Vamp4* | Vesicle-associated membrane protein 4 | – 1.38 |
|  | Mm.1408 | *Adm* | Adrenomedullin | – 1.46 |
|  | Mm.245715 | *Snap23* | Synaptosomal-associated protein 23 | – 1.52 |
|  | Mm.246753 | *Rab27b* | RAB27b | – 2.04 |
|  | Mm.302793 | *Syt9* | Synaptotagmin IX | – 2.25 |
